# Occupancy Models for Monitoring Marine Fish: A Bayesian Hierarchical Approach to Model Imperfect Detection with a Novel Gear Combination

**DOI:** 10.1371/journal.pone.0108302

**Published:** 2014-09-25

**Authors:** Lewis G. Coggins, Nathan M. Bacheler, Daniel C. Gwinn

**Affiliations:** 1 National Marine Fisheries Service, Southeast Fisheries Science Center, Beaufort, North Carolina, United States of America; 2 United States Fish and Wildlife Service, Yukon Delta National Wildlife Refuge, Bethel, Alaska, United States of America; 3 Biometric Research, and Program for Fisheries and Aquatic Sciences, School of Forest Resources and Conservation, University of Florida, Gainesville, Florida, United States of America; University of Siena, Italy

## Abstract

Occupancy models using incidence data collected repeatedly at sites across the range of a population are increasingly employed to infer patterns and processes influencing population distribution and dynamics. While such work is common in terrestrial systems, fewer examples exist in marine applications. This disparity likely exists because the replicate samples required by these models to account for imperfect detection are often impractical to obtain when surveying aquatic organisms, particularly fishes. We employ simultaneous sampling using fish traps and novel underwater camera observations to generate the requisite replicate samples for occupancy models of red snapper, a reef fish species. Since the replicate samples are collected simultaneously by multiple sampling devices, many typical problems encountered when obtaining replicate observations are avoided. Our results suggest that augmenting traditional fish trap sampling with camera observations not only doubled the probability of detecting red snapper in reef habitats off the Southeast coast of the United States, but supplied the necessary observations to infer factors influencing population distribution and abundance while accounting for imperfect detection. We found that detection probabilities tended to be higher for camera traps than traditional fish traps. Furthermore, camera trap detections were influenced by the current direction and turbidity of the water, indicating that collecting data on these variables is important for future monitoring. These models indicate that the distribution and abundance of this species is more heavily influenced by latitude and depth than by micro-scale reef characteristics lending credence to previous characterizations of red snapper as a reef habitat generalist. This study demonstrates the utility of simultaneous sampling devices, including camera traps, in aquatic environments to inform occupancy models and account for imperfect detection when describing factors influencing fish population distribution and dynamics.

## Introduction

Ecological surveys are important for understanding spatial and temporal variability in plant and animal populations, as well as providing the necessary feedback to guide policy options in the context of state-dependent and adaptive-management programs [1]. Individuals of a population are often counted directly in order to draw inferences about their abundance and distribution, but rarely are all individuals observed [2]. When the detection of individuals is imperfect, some portion of the population will be missed leading to erroneous conclusions and possibly erroneous management [3], [4], [5], [6]. Many ecological surveys instead use count or capture-rate data to index abundance. The implicit, and often violated, assumption of abundance indices is that capture probability does not vary systematically across space, time, habitat types, or environmental conditions [7], [8]. An alternative approach is to explicitly account for imperfect detection in sampling methodologies. Plot, distance, capture-recapture, and removal methods have all been used in terrestrial and aquatic environments to estimate animal abundance while accounting for imperfect capture probabilities [9], but these approaches are often impractical or expensive for many species [10].

The use of occupancy models to describe the distribution of populations while accounting for imperfect detection has increased in popularity over the last decade. These models require repeated sampling at spatially replicated sites to simultaneously estimate occupancy and detection probability, thereby correcting for imperfect detection [11], [4], [12]. Although the occurrence of a species at a site describes a different population process than abundance, occupancy models can be structured to estimate abundance directly by making some structural assumptions about the relationship between detection and abundance [3]. One major advantage of occupancy models for estimating population distribution and abundance is the use of incidence data that are often less costly to collect than data to estimate abundance directly (e.g., tagging information). Thus, occupancy models are gaining popularity in the wildlife literature as a monitoring tool.

Examples of occupancy modeling to index abundance or distribution are currently sparser in the fisheries literature than the wildlife literature. One reason for this discrepancy is the difficulties in sampling fish populations in ways that meet the assumptions of the model, particularly in marine systems, but see [13], [14], [9]. Because sampling fish is often invasive (e.g., electrofishing, trawling), replicate samples may not meet the assumption of independence [15] as the first sample may affect the detection of fish in the following samples. Temporal replicates that allow enough time between samples for the fish to recover from previous handling can be employed, but increase the risk of violating the population closure assumptions [11], [16]. Spatial replication of fish sampling at a site is often employed to ameliorate this issue; however, substituting spatial for temporal replicates can cause bias in parameters estimates under common sampling schemes [17], [18]. These sampling issues put fisheries managers at a great disadvantage because fisheries indices of abundance that inform policy choices are known to be plagued with issues of detection [19], [20], [21]. The lack of account for these detection issues has, in some cases, lead to inappropriate management choices and devastating ecological and economic losses, e.g., [22], [23]. For example, the collapse of the North Atlantic cod stock in 1992 is cited as one the greatest social and economic tragedies in Canada’s history and is partially attributed to a systematic increase in detection probability that caused abundance indices to remain stable as the stock declined [24]. Thus, methods that allow fisheries researchers to account for incomplete or variable detection when estimating the abundance and distribution of stocks are paramount.

An alternative sampling scheme to achieve temporal and spatial replication is the use of multiple sampling gears simultaneously. The use of multiple gears with occupancy models is uncommon in the ecological literature, but has been utilized to expand detection opportunities across species and individuals with variable vulnerability to different detection methods [25], [26]. Additionally, multiple gears employed in a nested design have been used to estimate occupancy probability at different spatial scales [27]. The use of non-invasive sampling gears such as camera traps in combination with traditional fish sampling [28], [29] may resolve some issues associated with replicate sampling described above. With replicate observations from simultaneous deployment of different gears in time and space, issues of bias induced by closure violations and non-independence of individual detections may be avoided. Thus, sampling with multiple gears combined with occupancy models may represent a powerful tool for monitoring fish populations.

Red snapper *Lutjanus campechanus* along the southeast USA coast (SEUS) is an economically important marine fish species and their management would benefit greatly from basic knowledge regarding their abundance and distributional patterns [30]. Since 2010, the SEUS red snapper fishery has been closed due to overfishing, and the only long-term survey data that exist (i.e., chevron trapping) have been deemed unusable in recent stock assessments due to overdispersed catches, as well as the perceived low detection probability [31], [32]. Beginning in 2010, high-definition video cameras have been attached to chevron traps to presumably increase gear detection probability [28]. Thus, the addition of cameras to the existing monitoring program is deemed critical for recovering and sustainably managing the red snapper fishery.

Here we develop occupancy and abundance models that employ incidence data collected with a combination of traditional invasive (chevron trap) and non-invasive (camera trap) sampling gears. This gear combination is novel to marine fisheries research and allows for a gear-for-time substitution for generating replicate samples that we expect will better meet the required assumptions of binomial sampling. Our specific objectives are to demonstrate the utility of these sampling methods by, 1) evaluating the relative fit of models that obtain sample replication from a combination of chevron trap and aggregated and disaggregated camera trap data, and 2) apply these models to evaluate how time and habitat influence red snapper occupancy probability and abundance as well as how gear and habitat influence detection.

## Materials and Methods

### Ethics Statement

Data collection for this study was authorized in a 5-year Scientific Research Permit (that commenced in 2010), issued by the Administrator of Southeast Regional Office of the National Marine Fisheries Service, National Oceanic and Atmospheric Administration, United States Government. This Scientific Research Permit covered all areas sampled in the study. All research followed the guidelines of the U.S. Government Principles for the Utilization and Care of Vertebrate Animals Used in Testing, Research, and Training (http://grants.nih.gov/grants/olaw/references/phspol.htm#USGovPrinciples). Red snapper collected in fish traps were euthanized by being placed on ice, after which a variety of biological samples were extracted per the guidelines of the Scientific Research Permit.

### Sampling Program

**Figure 1:**
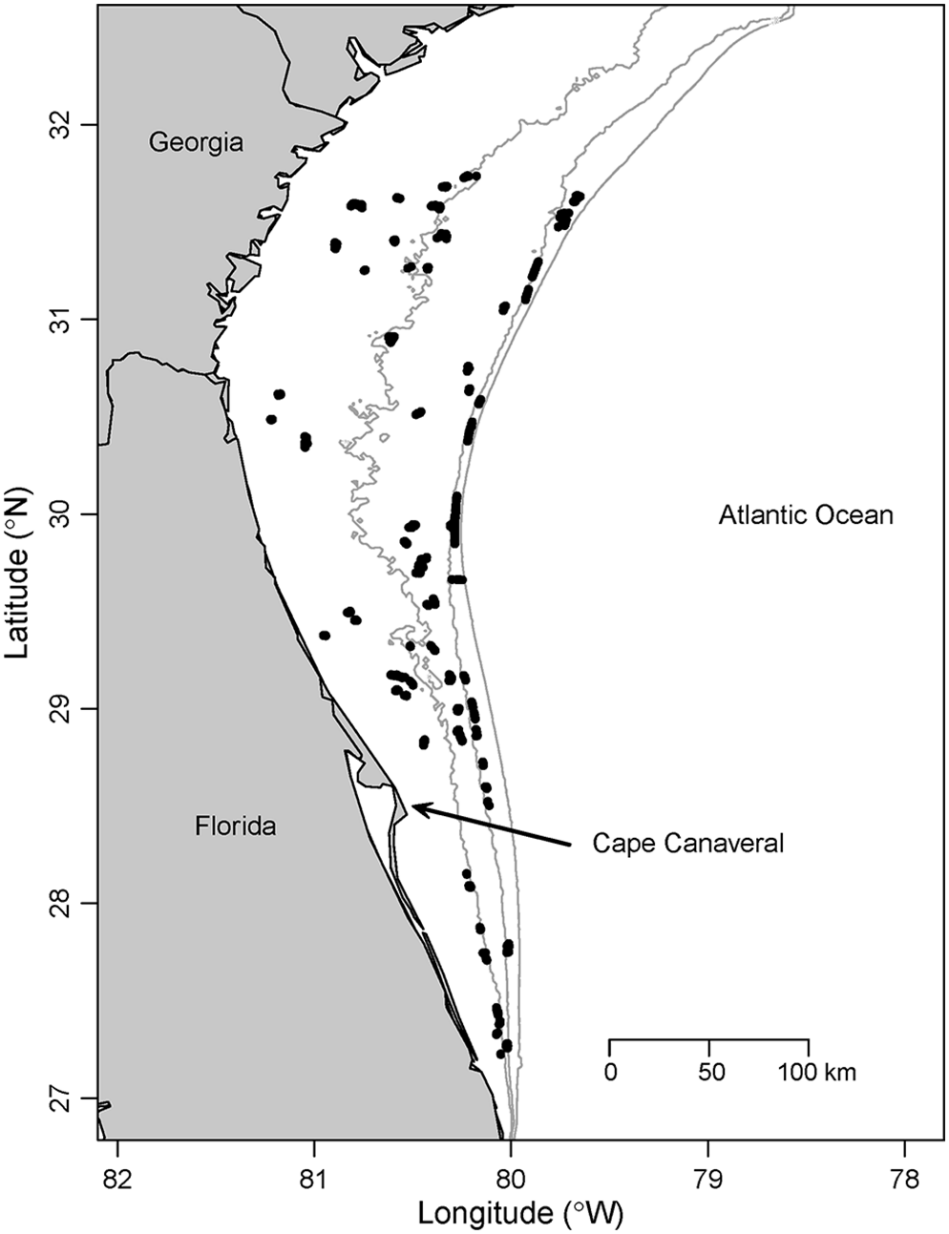
Study area with dots marking sampling locations. The offshore contours are depth isoclines at 30 m, 50 m, and 100 m.

Sampling occurred in Atlantic Ocean continental shelf waters off Georgia and Florida, USA, which encompass the historical center of the red snapper fishery in the SEUS [31] (Figure 1). Sampling targeted red snapper and other reef fishes that typically associate with hard substrates, which occur as scattered patches within the dominant sand and mud substrate of the region [33]. Patches of hard substrates in the SEUS are diverse and consist of flat limestone pavement, ledges, rocky outcroppings, or reefs, and are often colonized by various types of attached biota [34], [35]. The major oceanographic feature of the SEUS is the Gulf Stream, which influences outer sections of the continental shelf as it flows northward.

Sampling was conducted by the Southeast Fishery-Independent Survey (SEFIS), a fishery-independent sampling program created by the National Marine Fisheries Service in 2010 to increase fishery-independent sampling in the SEUS. Hard bottom sampling sites were selected for sampling in one of three ways. First, most sites were randomly selected from a sampling frame of hard bottom sampling points developed by SEFIS or the Marine Resources Monitoring, Assessment, and Prediction program of the South Carolina Department of Natural Resources. Second, some sites were sampled opportunistically even though they were not randomly selected for sampling in a given year. Third, new sites were added during the study period using information from fishermen, charts, and historical survey information. These locations were investigated using the vessel echo sounder and sampled if hard bottom was suspected to be present. Overall, less than 10% of the sampled sites included in the study were selected non-randomly via the second and third methods above. All sampling for this study occurred in 2010–2011 during daylight hours aboard the R/V *Savannah*, NOAA Ship *Nancy Foster*, or NOAA Ship *Pisces*. Depths ranged from 16 to 83 m.

**Figure 2:**
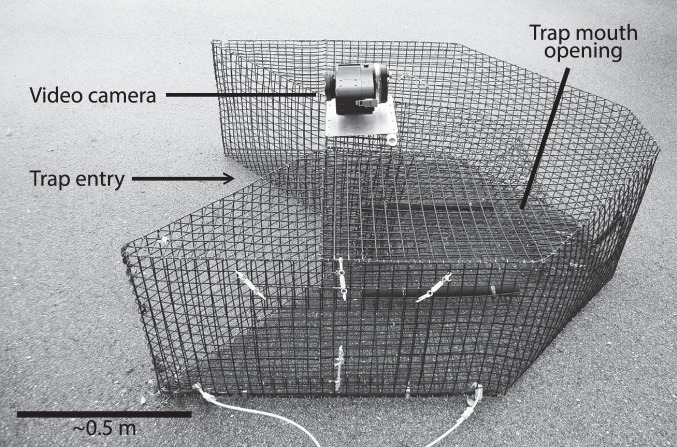
Chevron fish trap outfitted with an outward-looking Canon high-definition video camera over the mouth of the trap.

Chevron/camera traps were deployed at each selected site and consisted of a chevron trap outfitted with an outward-looking high-definition video camera (Figure 2). Eighteen to 24 trap combinations were deployed for approximately 90 min each day during April-October of each year. Traps were always spaced more than 200 m apart and each chevron trap was baited with 24 menhaden *Brevoortia* spp. Chevron traps have been used widely to index the abundance of reef fish and invertebrate species [36], [37], [38]. Chevron traps were constructed from plastic-coated galvanized 12.5-ga wire (mesh size = 3.4 cm^2^), and were shaped like an arrowhead measuring 1.7 m×1.5 m×0.6 m, with a total volume of 0.91 m^3^ (Figure 2).

A GoPro Hero (2010) or Canon Vixia HFS200 (2011) camera was attached over the mouth of each chevron trap. This camera positioning allowed for the potential detection of fish that are available to be caught by the chevron trap whether they actually enter or do not enter the trap. These cameras have similar viewing areas and resolution, and we assumed that camera type did not influence detection probability. We examined 1-second “snapshots” every 30 seconds beginning 10 minutes after the chevron trap was deployed and continuing for 20 minutes (for a total of 41 snapshots; [39]). If red snapper were seen in any of the 41 snapshots, they were considered present in the camera trap. This sampling strategy allowed us to gain replication at each sampling site from the simultaneous use of the chevron trap sample and the aggregate sample of the camera, or gain additional replication by using the disaggregated 41 1-second snapshots from the camera.

### Hypothesized Predictors of Occurrence and Detection

Red snapper site occurrence is likely influenced by latitude, depth, temperature, and various localized reef characteristics associated with substrate type and bottom topography [40], [41]. Habitat features such as substrate relief (i.e., the amount of topographic variation), percent hard bottom (i.e., substrate consisting of consolidated sediments), and percent attached biota (i.e., substrate with attached coral or sponges) are potentially important drivers of habitat suitability. Latitude, depth, and temperature are also likely to influence occurrence at broader spatial scales via processes potentially related to the native range of the species, the probability of influence by warm Gulf Stream currents, or fishery exploitation patterns.

Covariates that influence the observation process may be shared or unique to each gear type. Chevron trap detection probability is possibly related to deployment time (influencing the probability of fish encountering the trap), current speed (influencing the intensity and area of the bait plume scent), temperature (influencing fish activity or metabolism), and current direction (influencing how fish might orient to the trap). The camera trap detection process is possibly influenced by water clarity (limiting the detection range) and current direction and speed, which may affect the orientation or staging location of fish relative to the camera orientation.

We only analyzed sites that contained both valid chevron and camera trap samples. Sites were excluded if the chevron trap bounced or drifted, the chevron trap mouth opening was blocked by rocks, the video was dark, or any video files were missing. Year, latitude, longitude, depth, and bottom temperature were recorded at each sampling station. Bottom temperature (°C) was determined using a Seabird “Conductivity, Temperature, and Depth” instrument package (CTD; model SBE 25, Bellevue, Washington, USA). CTD casts were conducted near the middle of each sampling period, and the instrument was lowered to within 2 m of the bottom. Underwater videos were used to determine microhabitat features, water clarity, and current direction and magnitude around the chevron/camera trap (Table 1). The data analyzed in this study are reported in Table S1.

**Table 1:** Variables evaluated as potential covariates influencing patterns in occurrence, abundance, and/or detection of red snapper *Lutjanus campechanus*.

Covariate	Description
*Yr2011*	Data collected in 2011
*depth*	Depth of water in meters
*depth^2^*	Depth squared
*lat*	Latitude of sample site in decimal degrees
*lat^2^*	Latitude squared
*temp*	Bottom temperature (°C) of water at sample location
*temp^2^*	Bottom temperature squared
*livebot.l*	0–10% of substrate covered by live bottom (e.g., corals, sponges)
*livebot.m*	11–40% of substrate covered by live bottom
*livebot.h*	>40% of substrate covered by live bottom
*hardsub.l*	0–10% of substrate is hard bottom (e.g., rocks, boulders, ledges)
*hardsub.m*	11–40% of substrate is hard bottom
*hardsub.h*	>40% of substrate is hard bottom
*relief.m*	Maximum topographical relief of substrate is 0.3–1.0 m
*relief.h*	Maximum topographical relief of substrate is >1.0 m
*soak*	The total amount of time the trap was deployed (minutes)
*cdir.p*	Current direction is perpendicular to the trap mouth opening
*cdir.a*	Current direction is away from the trap mouth opening
*cspeed*	Speed of the current (low or high)
*turb.h*	Indicates high turbidity (i.e., cannot see bottom habitat)

Covariates not listed here (e.g., *yr2010*) inform the models’ intercept predictions.

### Modeling Overview

We modeled the occurrence of red snapper within a Bayesian hierarchical framework using a distribution sub-model that described how organisms are distributed among sites and a detection sub-model that described the data-generating process [42]. We also extended the occupancy model to account for variation in detection probability due to variation in fish abundance among sites using methods developed by Royle and Nichols [3]. This model formulation assumes a relationship between the probability of detecting a species and the number of individuals of that species at a site. This is accomplished by estimating the probability of detecting a single individual (individual-based detection). The advantage of modeling abundance is that variation in the probability of detecting a species due to abundance variation among sites is explicitly accounted for and covariates are evaluated for effects on the abundance of fish. We evaluated performance of six candidate models including both the basic occupancy model [11] and the abundance model (Royle-Nichols [3]) formulation with both distribution and detection model covariates, with and without random effects on the detection process, and with aggregated and disaggregated detection histories for the camera trap (Table 2). Additionally, we fit the Royle-Nichols formulation both with and without random effects on mean site abundance.

**Table 2:** General model structures evaluated for convergence properties and goodness of fit (GOF).

Model Type	Model Structure	Camera Trap Data	G-R	GOF
**Basic Occupancy**	**Bin(** ***ψ*** **) Bin(** ***p_chevron_*** **) Bin(** ***p_camera_*** **)**	**pooled**	**1.0**	**0.99**
Basic Occupancy	Bin(*ψ*) Bin(*p_chevron_*) Bin(*p_camera_*)	disaggregated	1.0	0.00
Basic Occupancy	Bin(*ψ*) Bin(*p_chevron_*) Bin-logNorm(*p_camera_*)	disaggregated	1.0	0.00
**Royle-Nichols**	**Pois(** ***λ*** **) Bin(** ***p_chevron_*** **) Bin(** ***p_camera_*** **)**	**pooled**	**1.0**	**0.71**
Royle-Nichols	Pois(*λ*) Bin(*p_chevron_*) Bin(*p_camera_*)	disaggregated	1.0	0.00
Royle-Nichols	Pois-logNorm (*λ*) Bin(*p_chevron_*) Bin(*p_camera_*)	disaggregated	1.0	0.00

The camera trap data column indicates models using pooled detections versus disaggregated detections. Values of GOF approaching zero indicate lack of fit while values approaching one indicate no evidence of lack of fit. Values of the Gelman-Rubin statistic (G-R) close to one indicate model convergence while values greater than one indicate lack of model convergence. Bolded models converged and displayed no evidence of lack of fit.

### Basic Occupancy Model Structure

We defined occurrence as *z_i_* where *z* is a binary variable indicating the latent occupancy state of red snapper at site *i* with *z* = 1 indicating presence and *z* = 0 indicating absence. We assumed that *z_i_* was the result of a Bernoulli trial represented by *z_i_* ∼Bernoulli(*ψ_i_*), where *ψ_i_* represents the probability of occurrence of red snapper at site *i*. Because the true occupancy state is observed imperfectly, we modeled the probability of detection of red snapper as a separate Binomial process for each gear, where the unconditional probability of detection at site *i* with gear *j* is *z_i_ p_ij_*. Thus, the number of observations of red snapper is represented as 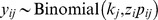, where *p_ij_* is the detection probability that is conditional on *z_i_* = 1, and *k* indicates the number of replicate samples collected by each gear at each site.

We incorporated potential covariate effects in the distribution sub-model using a logit link [43] specified as:
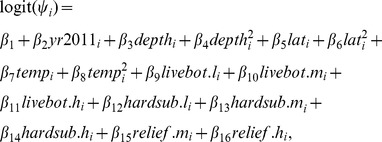
(1)where *β*_1_ represents the intercept of the distribution sub-model and *β*_2_, *β*_3_,….*β*_16_ represent the logit-scale effects of each variable on the probability of the occurrence. Variables and their abbreviations are defined in Table 1.

A detection probability sub-model was developed for each gear type. We specified the chevron trap detection probability *p_ij_*_ = 1_ as:
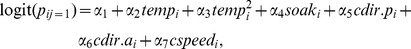
(2)where *α*_1_ represents the intercept and *α*_2_, *α*_3_,……*α*_7_ represent the logit-scale effects of each covariate on detection probability (Table 1). We specified camera trap detection probability *p_ij_*_ = 2_ as:
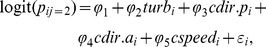
(3)where *φ*_1_ represents the intercept and *φ*_2_ through *φ*_5_ represent the logit-scale effects of each variable on the probability of detection by the camera trap (Table 1). The parameter *ε_i_* is a site-specific random effect to account for possible variation in detection probability among sites not explained with covariates (see Table 2). We modeled *ε_i_* as a normally distributed random variable with mean equal to zero and standard deviation σ_ε_. The random effects model structure was only used when considering the disaggregated camera trap data containing sufficient information to assure all model parameters were identifiable.

### Royle-Nichols Model Structure

We modeled the abundance of red snapper based on methods first proposed by [3] and then extended into a hierarchical framework by [42]. Because we cannot observe the abundance at sites directly, we specified site abundance 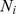 as a latent random effect with variation that is explained by a Poisson distribution across *i* sites as *N_i_* ∼Poisson(*λ_i_*), where *λ_i_* represents the mean site abundance. Our data 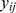 are the frequencies of red snapper detections at site *i* with gear *j*. We assume that 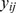 is the result of binomial outcomes as 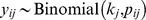, where 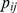 is the probability of detecting at least one individual at site *i* with gear *j* and 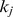 is the number of replicate samples collected with gear *j*. We linked the distribution sub-model to the detection sub-model by specifying the relationship between 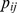 and 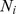, per [3], as 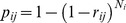 where 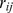 is individual-based detection probability, as opposed to 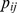, which is the probability of detecting at least one individual at site *i* with gear *j*. This formulation essentially models the detection probability 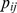 as a random effect due to variation in fish abundance among sites.

We utilized a similar covariate structure for the of the Royle-Nichols distribution sub-model as we did for the basic occupancy model with log of mean site abundance (*λ_i_*) specified as:
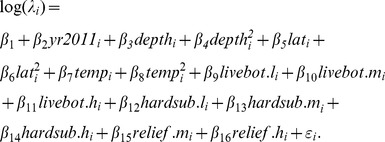
(4)


The parameter *ε_i_* was a site-specific random effect drawn from a Normal distribution with mean of zero and standard deviation (σ_ε_) to account for extra-Poisson variation in abundance across sites. Similar to the basic occupancy model structure, the random effects model was only fit to the disaggregated camera trap data to assure all parameters were identifiable. Covariates were incorporated into the detection sub-models with a logit link as:
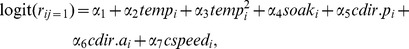
(5)

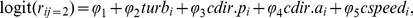
(6)where *r_ij_*_ = 1_ is the individual detection probability for chevron traps and *r_ij_*_ = 2_ is the individual detection probability for camera traps. All continuous variables were centered on the mean and scaled by one standard deviation to help with fitting and allow for unambiguous comparison of parameter estimates.

### Model Evaluation

We evaluated the performance of six candidate models to determine the model structure that best described the data (Table 2). These models included both the basic occupancy model and the Royle-Nichols model fit with camera trap detection models employing both aggregated and disaggregated detection data. The aggregated camera trap detections used the entire 41 snap-shots as one sample while the disaggregated camera trap detections considered each snap-shot as an independent sample to determine if there was a trade-off between the number of replicates and effort per replicate that may optimize model fit. We hypothesized that more replicates would better inform estimates of the detection process than a single aggregated sample. However, disaggregated detections may sacrifice model fit because of auto-correlation among the snap shots. For the basic occupancy models and considering the disaggregated camera trap data, we evaluated model structures with and without site-specific random effects on the detection process of the camera traps to account for variation in detection that was not explained by the site-specific covariates (3). For the Royle-Nichols models and considering the disaggregated camera trap data, we evaluated model structures with and without site-specific random effects on abundance in the distribution sub-model to account for extra-Poisson variation in abundance (4). This model extension, in turn, modeled additional variation in the detection process of both the cheveron trap and the camera trap through the relationship between detection probability and abundance.

We performed our model evaluations at two different levels. First we evaluated model fit with a Chi-squared goodness of fit (GOF) test developed by [44]. Although lack of fit can result for many different reasons, adequate fit of a model would indicate that the assumptions of the model such as independent detections among camera snap shots or between the camera and the chevron trap were adequately met. The GOF test is a form of posterior predictive check that assumes the frequency of detection histories conform to a Chi-square distribution. Point estimates of all parameter values from the full model are used to perform a parametric bootstrap to determine the expected distribution of model deviances. The deviance of the observed detection frequencies is then compared to the distribution of expected deviances to determine model fit. Goodness of fit values range from zero to one with values approaching zero indicating lack of fit and values approaching one indicating adequate fit.

For all models judged to fit adequately by the GOF [44] procedure, we further evaluated the support of each covariate as our second level of model evaluation. We considered all combinations of covariates plausible and estimated the inclusion probability of each covariate using a mixture modelling approach where each covariate is multiplied by an “inclusion parameter” ([42], pages 72–73). The inclusion parameters (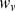 for all variables in the model) were latent binary variables with uninformative prior probabilities of 0.5 (i.e., equal probability model inclusion or exclusion). The posterior probabilities of the inclusion parameters correspond to the probability that the given variable is included in the model, and parameter summaries and predictions are averaged across all covariate combinations. Following [45], we assumed that parameters with inclusion probability greater than 50% were adequately supported by the data.

Posterior probability distributions of model parameters were estimated using a Monte Carlo-Markov chain (MCMC) algorithm implemented in program JAGS [46]. We called JAGS from within program R [47] with the library RJAGS (http://mcmc-jags.sourceforge.net). All prior distributions were uninformative distributions specified to have little influence on the posterior probability distributions. The prior distributions of all logit-scale parameters were specified as *t*-distributions with σ  = 1.566 and ν  = 7.763 per [48] such that back-transformed values assigned equal probability for all values between zero and one. Priors of standard deviation parameters were specified as uniform distributions with equal probability between zero and 100 and were verified to not influence the range of the posterior distributions. Inference was drawn from 30,000 posterior samples taken from 3 chains of 100,000 samples thinned to every 10. We allowed a burn in of 10,000 samples to remove the effects of initial values. Convergence was diagnosed for the full model by visual inspection of the MCMC chains for adequate mixing and stationarity and by using the Gelman-Rubin statistic (with values <1.1 indicating convergence; [49]).

## Results

The Gelman-Rubin statistic indicated that all of the models converged to stable posterior distributions (Table 2). Based on the [40] GOF statistic, all of models considering disaggregated camera trap data exhibited poor fit. Inspection of the raw incidence data suggests that the poor fit was largely influenced by a small number of sites with much higher camera trap detection frequencies than predicted by the model structure and covariates considered. The remaining two models exhibiting good fit considered the pooled data and did not include log-normal random effects on either abundance or camera trap detection. We present detailed results from both of these models, Bin(*ψ*) Bin(*p_chevron_*) Bin(*p_camera_*) and Pois(*λ*) Bin(*p_chevron_*) Bin(*p_camera_*), and compare and contrast the basic occupancy versus the Royle-Nichols models. The computer code for these models is reported in Appendix S1 and S2.

The occupancy probability across years was estimated as 0.45 (95% CI = [0.30, 0.57]) for the basic occupancy model and 0.48 (95% CI = [0.32, 0.61]) for the Royle-Nichols model. As expected, the observed occupancy rates, uncorrected for imperfect detection, were lower for both the chevron trap (0.14) and camera trap (0.31) data. The estimated mean abundance (*λ*) across sites from the Royle-Nichols model ranged between 0.01 and 2.08 fish site^−1^. In contrast, the observed chevron trap catch across sites ranged between 0 and 13 fish site^−1^ and the observed maximum camera trap count ranged between 0 and 14 fish site^−1^. While maximum numbers of fish observed per site are larger for the chevron and camera trap than the maximum estimated *λ*, the proportion of sites where the observed numbers of fish was greater than 2 was small for both the chevron (0.04) and the camera (0.07) trap data.

### Distribution

**Figure 3:**
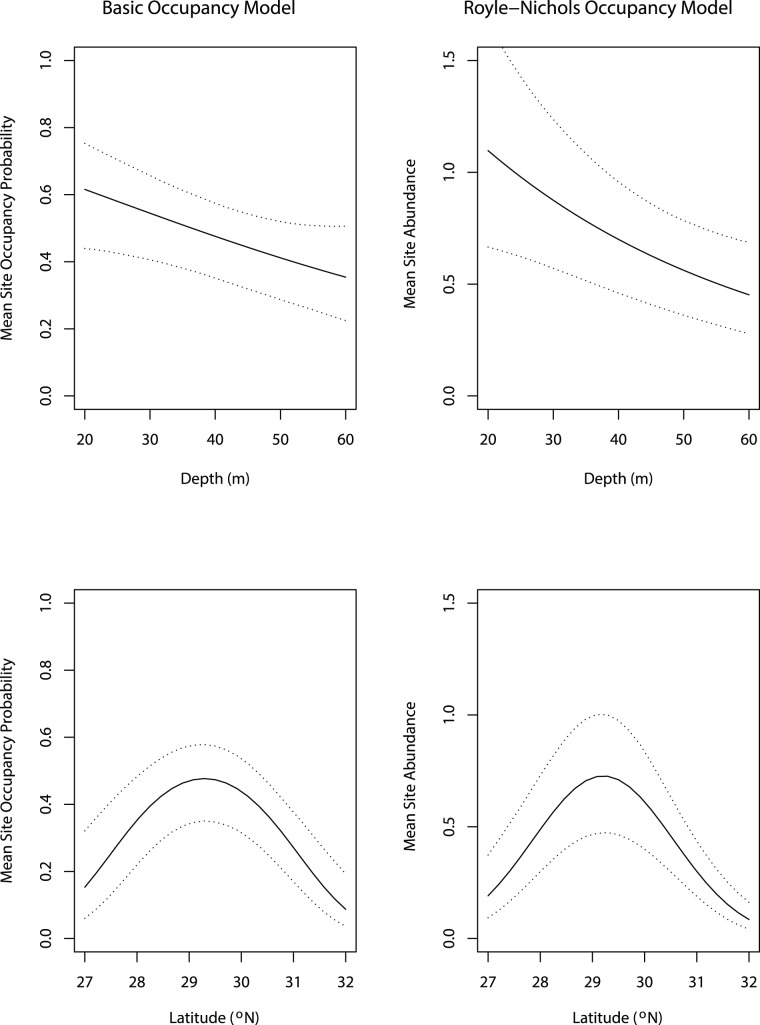
Mean site occupancy probability and abundance of red snapper as a function of depth and latitude. The left column contains the estimated mean site occupancy probability as a function of depth (top panel) and latitude (bottom panel) from the basic occupancy model. The right column contains the estimated mean site abundance as a function of depth (top panel) and latitude (bottom panel) from the Royle-Nichols occupancy model. The intervals represent 95% credible intervals.

The posterior inclusion probabilities for the basic occupancy model indicated that the distribution sub-model best supported by the data included the covariates: water depth (*depth*, Pr = 0.87; Table 3), latitude (*lat*, Pr = 0.95), and its squared term (*lat^2^*, Pr = 0.99). This model predicted that occupancy probability should vary between approximately 0.6 and 0.3 with a depth change from 20 m to 60 m (Figure 3). The model also predicted that occupancy probability should vary quadratically with latitude peaking (∼0.48) at approximately 29.5°N and declining to near 0.2 at 27.2°N and 31.3°N. Notably, the year covariate (*yr2011*) was not supported by the data, suggesting no temporal trend in the occurrence probability of red snapper between 2010 and 2011.

**Table 3:** Posterior probability summaries of parameters evaluated in the red snapper *Lutjanus campechanus* basic occupancy model using pooled camera trap data and without a site-specific random effect on camera trap detection probability (Bin(*ψ*) Bin(*p_chevron_*) Bin(*p_camera_*)).

			Credible interval		Inclusion
Parameter	Mean	SD	2.5%	97.5%	probability
Distribution model					
* intercept*	−0.188	0.281	−0.844	0.279	−
* yr2011*	−0.003	0.075	−0.166	0.082	8%
* * ***depth***	−**0.330**	**0.167**	−**0.602**	**0.000**	**87%**
* depth^2^*	0.013	0.055	0.000	0.215	8%
* * ***lat***	−**0.401**	**0.155**	−**0.672**	**0.000**	**95%**
*** lat^2^***	−**0.421**	**0.105**	−**0.624**	−**0.220**	**99%**
*** *** *temp*	0.007	0.047	0.000	0.158	6%
*** *** *temp^2^*	0.000	0.018	0.000	0.000	3%
* livebot.l*	0.153	0.288	0.000	0.957	30%
* livebot.m*	0.044	0.178	−0.045	0.684	14%
* livebot.h*	−0.307	0.401	−1.202	0.000	46%
* hardsub.l*	0.070	0.216	0.000	0.799	17%
* hardsub.m*	0.126	0.276	0.000	0.938	25%
* hardsub.h*	0.005	0.163	−0.333	0.365	11%
* relief.m*	0.008	0.080	−0.023	0.235	8%
* relief.h*	−0.055	0.194	−0.726	0.011	16%
Chevron trap detection model					
* intercept*	−0.499	0.184	−0.876	−0.155	−
* temp*	−0.055	0.130	−0.455	0.000	20%
* temp^2^*	0.003	0.036	0.000	0.056	5%
* soak*	−0.005	0.043	−0.106	0.000	6%
* cdir.p*	0.008	0.103	−0.105	0.280	9%
* cdir.a*	0.014	0.123	−0.130	0.392	10%
* cspeed*	0.051	0.410	−0.769	1.249	21%
Camera trap detection model					
* intercept*	1.126	0.533	0.132	2.213	−
*** turb.h***	**0.559**	**0.639**	**0.000**	**1.920**	**55%**
* cdir.p*	−0.067	0.332	−1.082	0.477	20%
*** cdir.a***	**0.652**	**0.783**	**0.000**	**2.405**	**54%**
* cspeed*	−0.034	0.642	−1.595	1.571	28%

All metrics were calculated from model averaged posterior distributions using the Bayesian mixture modeling approach. Bolded parameters had inclusion probability greater than 50%.

The distribution sub-model of the Royle-Nichols model produced similar results to the basic occupancy model likely due to both low site-specific true abundances and little variation in true abundance among sites. Water depth (*depth*, Pr = 0.96; Table 4) and both latitude (*lat*, Pr = 1.00) and its squared term (*lat^2^*, Pr = 1.00) were the most important covariates influencing abundance in the model (Table 4). On average, predicted abundance ranged between 1.0 and 0.5 individuals per site as depth changed from 20 m to 60 m (Figure 3). Abundance was also predicted to have a quadratic relationship to latitude, with a predicted abundance of about 0.20 individuals per site at 27°N and 31.5°N and peaking at approximately 0.75 individuals per site at 29.5°N (Figure 3). The Royle-Nichols model indicated that high coverage of the substrate with biota had a negative influence on abundance (*livebot.h*, Pr = 0.55; Table 4). This effect predicts a decrease of about 0.66 individuals per site between low (0–10%) and high levels (>40%) of live bottom coverage. The basic occupancy model suggested a similar effect, though the inclusion probability was slightly less than the 0.5 standard for inclusion. As with results of the basic occupancy model, mean abundance did not appear to differ between 2010 and 2011.

**Table 4:** Posterior probability summaries of parameters evaluated in the red snapper *Lutjanus campechanus* Royle-Nichols occupancy model using pooled camera trap data and without a site-specific random effect on abundance (Pois(*λ*) Bin(*p_chevron_*) Bin(*p_camera_*)).

			Credible interval		Inclusion
Parameter	Mean	SD	2.5%	97.5%	probability
Distribution model					
* intercept*	−0.433	0.222	−0.954	−0.066	-
* yr2011*	−0.006	0.060	−0.183	0.070	10%
*** depth***	−**0.277**	**0.101**	−**0.453**	**0.000**	**96%**
* depth^2^*	0.002	0.024	0.000	0.063	6%
*** lat***	−**0.414**	**0.106**	−**0.627**	−**0.214**	**100%**
*** lat^2^***	−**0.375**	**0.074**	−**0.525**	−**0.234**	**100%**
* temp*	0.006	0.036	0.000	0.127	8%
* temp^2^*	0.001	0.016	0.000	0.007	4%
* livebot.l*	0.140	0.228	0.000	0.744	37%
* livebot.m*	0.054	0.165	−0.081	0.589	21%
*** livebot.h***	−**0.281**	**0.318**	−**0.941**	**0.000**	**55%**
* hardsub.l*	0.037	0.134	−0.022	0.485	16%
* hardsub.m*	0.098	0.196	0.000	0.648	30%
* hardsub.h*	0.013	0.125	−0.199	0.373	15%
* relief.m*	0.027	0.094	−0.005	0.350	15%
* relief.h*	−0.042	0.147	−0.532	0.077	19%
Chevron trap detection model					
* intercept*	−0.940	0.217	−1.391	−0.542	-
* temp*	−0.083	0.150	−0.481	0.000	31%
* temp^2^*	0.005	0.049	−0.039	0.149	9%
* soak*	−0.011	0.058	−0.207	0.000	10%
* cdir.p*	0.012	0.136	−0.262	0.407	16%
* cdir.a*	0.037	0.170	−0.204	0.569	19%
* cspeed*	0.062	0.445	−0.899	1.252	32%
Camera trap detection model					
* intercept*	0.512	0.483	−0.388	1.515	-
*** turb.h***	**0.470**	**0.525**	**0.000**	**1.595**	**59%**
* cdir.p*	−0.025	0.294	−0.840	0.646	26%
*** cdir.a***	**0.817**	**0.710**	**0.000**	**2.259**	**73%**
* cspeed*	−0.039	0.563	−1.397	1.322	36%

All metrics were calculated from model averaged posterior distributions using the Bayesian mixture modeling approach. Bolded parameters had inclusion probability greater than 50%.

**Figure 4:**
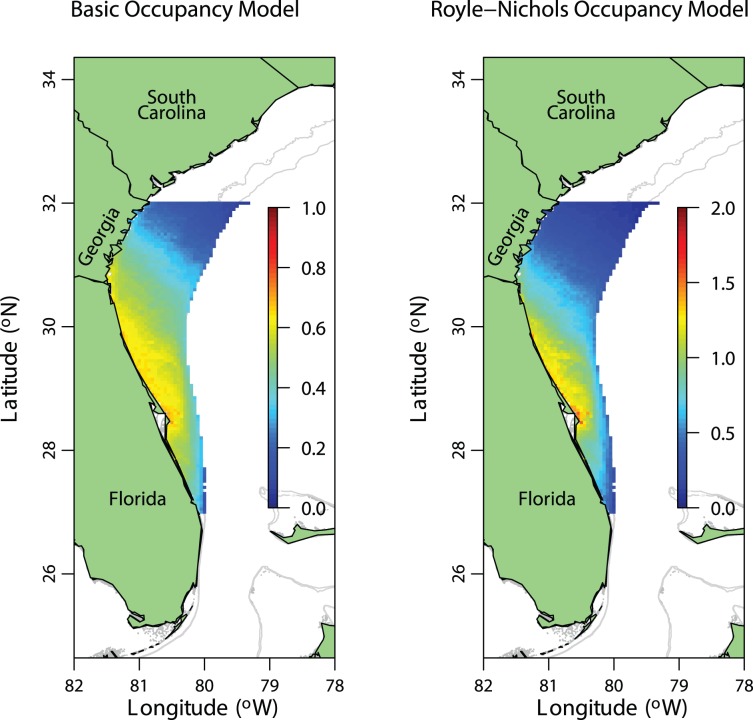
Mean site occupancy probability and abundance of red snapper in reef habitat off the coasts of Georgia and Florida. Estimated mean occupancy probability of reef sites from the basic occupancy model (left panel) and abundance from the Royle-Nichols model (right panel) as a function of depth and latitude.

We used the distribution sub-models from both the basic occupancy and Royle-Nichols models to predict both the occupancy probability and the mean abundance across a grid from approximately latitude 27°N to 32°N and from near-shore to a depth of 65 m (Figure 4). The model predicts that occupancy of reefs is highest in shallow waters (∼20 m) and between approximately Cape Canaveral (28.4°N) and the Georgia-Florida state boundary line. The Royle-Nichols model predicts highest abundance at reefs in similar locations. Both models predict the center of distribution to be located at reefs offshore from Cape Canaveral.

### Detection

**Figure 5:**
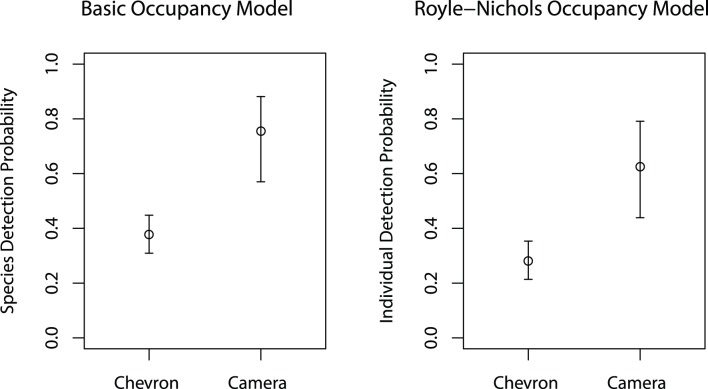
Mean chevron and camera trap detection probabilities. Estimated detection probabilities at the reference covariate values (intercept) from both the basic occupancy model (left panel) and the Royle-Nichols model (right panel) for red snapper. Note that for the basic occupancy model framework the detection probability is for the species at occupied sites. In contrast, for the Royle-Nichols framework the detection probability is for each individual at occupied sites. The intervals represent 95% credible intervals.

Our mixture model procedure indicated support for covariates of camera trap detection, but did not for covariates of chevron trap detection as all covariate inclusion probabilities were <0.50 (Tables 3 & 4). The largest effect on detection was between the chevron traps and the camera traps. Camera trap detection probabilities were predicted to be about twice the detection probabilities predicted for chevron traps for both the basic occupancy model and the Royle-Nichols model (Figure 5). Mean detection probabilities estimated with the basic occupancy models were similar in magnitude to mean individual-based detection probabilities estimated with the Royle-Nichols occupancy model, likely due to low mean site abundances. Mean species detection probability and individual-based detection probability values for the chevron traps were 0.39 and 0.30, respectively (Figure 5). For the camera traps, mean species detection probability and individual-based detection probability values were 0.75 and 0.61, respectively (Figure 5).

**Figure 6:**
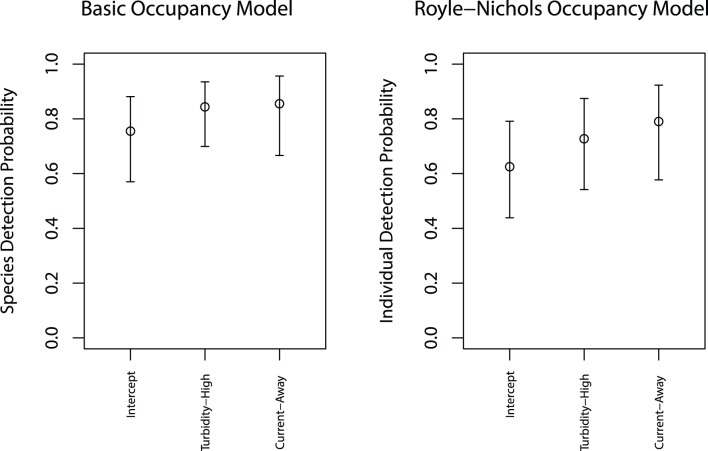
Camera trap detection probabilities as influenced by turbidity and current direction. The estimated detection probabilities at reference covariate values (intercept) and at high turbidity and with current direction away from the camera lens from both the basic occupancy model (left panel) and the Royle-Nichols model (right panel) for red snapper. Note that for the basic occupancy model framework the detection probability is for the species at occupied sites. In contrast, for the Royle-Nichols framework the detection probability is for each individual at occupied sites. The intervals represent 95% credible intervals.

Covariates found to influence camera trap detection were water turbidity and current direction and were consistent between the basic occupancy model and the Royle-Nichols occupancy model. The basic occupancy model predicted species detection probability to be 0.09 higher in turbid water than clear water (*turb.h*, Pr = 0.55, Table 3, Figure 6) and that mean detection probability to be at least 0.10 higher when the current direction was away from the camera lens (*dir.a*, Pr = 0.54). The results for individual-based detection probability (*r*) from the Royle-Nichols model were similar, but slightly stronger. The model predicted mean individual detection probability to be 0.10 higher in turbid water than clear water (*turb.h*, Pr = 0.59, Table 4) and mean individual-based detection probability to be at least 0.17 higher when the current direction was away from the camera lens (*dir.a*, Pr = 0.73).

## Discussion

We explored the utility of combining observations from novel camera trap methods with traditional chevron traps to inform occupancy models that characterize temporal and spatial patterns in reef fish distribution. While camera traps are occasionally combined with other detection methods in terrestrial studies [e.g., 50, 25], we are unaware of studies utilizing camera and fish traps to provide the replicate observations required to account for incomplete detection of marine fish. The use of multiple gears sampling simultaneously assures closure among replicate samples, which is a major advantage for informing occupancy models. This is particularly true in aquatic systems where the need to use invasive sampling methodologies can violate the closure assumption among temporal replications.

We initially hypothesized that the disaggregated camera trap data would be more informative than the pooled camera trap data because of larger numbers of replications. However, the models considering the disaggregated camera trap data exhibited poor fit and simple examination of the raw data suggested the presence of extra-binomial variation possibly caused by non-independence in detections among individuals at the same site. This potential violation of the binomial sampling assumption appears to limit the utility of the disaggregated data for both the basic and Royle-Nichols models. In particular, the Royle-Nichols model assumes detections are independent among individuals in the relationship between detection and abundance (*p* = 1−(1−*r*)*^N^*; [3], [51]). Violations of this assumption could occur if replicate samples are correlated or demonstrate extra-binomial variation. Correlation among samples could occur if camera trap snap shots were taken at too short of a time interval leading to serial auto-correlation. Alternatively, extra-binomial variation can occur when animals demonstrate characteristics that result in clustering as would be expected from schooling behavior in fish. Red snapper exhibit weak schooling behavior [52], which corroborates the evidence of lack of independence observed in our data.

The consequence of violating the binomial sampling assumption is a divergence from the relationship of occupancy and abundance [51] because groups of fish are perceived as individuals by the model. This violation results in an underestimation of abundance. Our analysis showed evidence of negative bias because the estimate of mean site abundance was less than the mean maximum count across sites. However, we expect little impact on the accuracy of occupancy probability estimates because occurrence is defined by the presence of one or more individuals. Furthermore, we expect the divergence of patterns in occurrence from patterns in abundance to be low because of low numbers of individuals per site, likely due to their overfished status in the SEUS [31].

Our findings suggested that depth and latitude explained most of the variation in red snapper occupancy rate and mean abundance among hard bottom sites. While this finding is clearly supported by the data and analysis, it is important to recognize that this does not imply that red snapper are not associated with reef habitat. Because nearly all samples considered in the analyses were collected on or near some amount of reef habitat, the analysis is incapable of describing the difference in occupancy probability or site abundance between hard bottom reef sites and non-reef sites composed entirely of unconsolidated sediments with little or no bottom relief. Instead, our analysis attempts to uncover differences among reefs with different amounts of hard bottom, attached biota, and topographic complexity. Our data supported only a weak and negative relationship between red snapper abundance and high incidence of attached biota. Overall this supports the existing evidence that red snapper may not strongly require specific reef characteristics and instead may be reef habitat generalists [40], [41]. However, caution must be practiced when extrapolating these results outside of our sample region, as a subset of our sites was selected non-randomly.

Both the basic occupancy and the Royle-Nichols models estimated the inclusion probability of the year effect (*yr2011*) to be much smaller than the 50% threshold. As such, the data do not support a positive or negative difference in either occupancy probability or site abundance between 2010 and 2011. Red snapper is currently under a management rebuilding plan to recover from a designated “overfished” status [31]. While our results do not imply a change in population between 2010 and 2011, we recognize that a longer time-series of data may be needed to observe any extant population trend [32]. However, evaluation of a temporal effect from occupancy models is potentially useful to both management and future stock assessments, particularly as more years of data become available.

A major benefit of analyzing the camera and chevron trap data in the occupancy modeling framework is to estimate both the species and individual detection probabilities for each gear. The model estimated that the camera-trap detection probability increased with higher turbidity and if the current direction was away from the camera lens. While we were not surprised that a current direction that tends to cause fish to orient themselves in the camera field of view would increase detection probability, we do not have a good explanation as to why high turbidity would favor detection over low turbidity. Perhaps red snapper tend to stage closer to the chevron and camera traps when the water is more turbid because they are visual predators and the chevron trap is perceived as structure; however, the effect was small indicating this would not be a pronounced behavior. The original motivation to collect video information during chevron trap deployments was to evaluate whether red snapper were frequently present near chevron traps but not captured. Our work suggests that camera traps are approximately twice as likely as chevron traps to detect red snapper. Additionally, at least one red snapper will be detected with >95% probability with the camera trap so long as ≥4 individuals are present. In contrast, ≥9 individuals must be present to have a 95% probability of detecting the species with the chevron trap.

We evaluated the use of occupancy style models that estimate patterns in occurrence and abundance [3] with incidence data. One limitation of these analyses for evaluating distributional patterns occurs for species that inhabit most sampling sites and therefore demonstrate little variation in occurrence rates. Under these conditions, occupancy probability is not an informative metric of population change. A further limitation occurs for species with high abundance at occupied sites such that the species is detected in most or all replicate samples. When this occurs, the Royle-Nichols model can produce negatively biased estimates of abundance. Although these were not the conditions for red snapper in the SEUS, they can be the condition for more ubiquitous or abundant species. For example, black sea bass *Centropristis striata* demonstrate high catches in the SEFIS data and would likely limit the utility of the Royle-Nichols model for generating unbiased estimates of abundance. Thus, occupancy models may perform best for species with some level of rarity such as red snapper, but may be a less useful monitoring tool for more ubiquitous or abundant species.

A natural extension of occupancy models not limited by high occurrence rates and high abundance is a class of models referred to as binomial-mixture models or *N*-mixture models [53]. Similar to the Royle-Nichols occupancy model, binomial-mixture models assume that abundance is distributed across sites according to a distribution (e.g. Poisson) and that catches are the result of replicated binomial processes. However, binomial-mixture models fit count data as opposed to incidence data, which can be more informative of the abundance and detection process. Research on the use of binomial-mixture models for monitoring fish is more limited than for occupancy models (but see [54]); however, some research has been done on the use of multiple sampling methods with binomial-mixture models of terrestrial species. For example, [24] applied multiple sampling methods in a binomial-mixture model of grizzly bears in Glacier National Park. [55] then investigated the conditions when it is appropriate to combine multiple sampling methods into a single binomial-mixture model for grizzly bears. Evaluating these methods for marine fish catch data generated with the combined chevron trap and camera trap described here would be a valuable contribution to the ecological literature.

Accounting for incomplete and variable detection of fish is rare in the ecological literature because invasive sampling methods inhibit appropriate replication. Our analysis demonstrates how the use of non-invasive camera traps can be paired with invasive fish traps to generate replicate samples needed to account for incomplete and variable detection for marine fish. This work has broad implications for fisheries management because fisheries data are notoriously expensive and plagued with issues of variable detection. Furthermore, the costs of inappropriate management of fish stocks are high for commercially and recreationally valued species. Thus, the methods we demonstrate here could improve the management of many exploited fish stocks and reduce the risks of economic and biodiversity loss.

## Supporting Information

Table S1 Data analyzed in this study. Note that the first two columns contain the numbers of red snapper detections (det) from the chevron and camera traps. Refer to Table 1 for a description of the covariate abbreviations.
(DOCX)

Appendix S1 JAGS code for basic occupancy model of red snapper occurrence and detection with pooled camera trap detections. The “#” symbol precedes annotation remarks. Refer to the Methods section of the main text for symbol equations and parameter definitions.
(DOCX)

Appendix S2 JAGS code for Royle and Nichols (2003) occupancy model of red snapper abundance and detection with pooled camera trap detections. The “#” symbol precedes annotation remarks. Refer to the Methods section of the main text for symbol equations and parameter definitions.
(DOCX)
