# Supplementary material for: Occupancy Models for Monitoring Marine Fish: A Bayesian Hierarchical Approach to Model Imperfect Detection with a Novel Gear Combination
Source: PLoS One. 2014 Sep 25;9(9):e108302. doi: 10.1371/journal.pone.0108302 (PMC4178000; doi:10.1371/journal.pone.0108302)
Supplement: Table S1 — Data analyzed in this study. Note that the first two columns contain the numbers of red snapper detections (det) from the chevron and camera traps. Refer to Table 1 for a description of the covariate abbreviations. (DOCX) [file pone.0108302.s001.docx]

| chevron .det | camera .det | year | depth | lat (scaled) | temp | lb .low | lb .med | lb .high | hs .low | hs .med | hs .high | relief .med | relief .high | soak | cdir .p | cdir .a | cspeed .high | turb .high |
| --- | --- | --- | --- | --- | --- | --- | --- | --- | --- | --- | --- | --- | --- | --- | --- | --- | --- | --- |
| 1 | 16 | 2010 | 54 | -0.98 | 27.4 | 1 | 0 | 0 | 0 | 0 | 1 | 0 | 1 | 165 | 1 | 0 | 0 | 0 |
| 0 | 0 | 2010 | 60 | -0.97 | 27.4 | 1 | 0 | 0 | 0 | 1 | 0 | 0 | 0 | 108 | 1 | 0 | 1 | 0 |
| 1 | 24 | 2010 | 52 | -0.97 | 27.4 | 1 | 0 | 0 | 0 | 0 | 1 | 0 | 0 | 96 | 1 | 0 | 1 | 0 |
| 0 | 0 | 2010 | 52 | -0.97 | 27.4 | 1 | 0 | 0 | 0 | 0 | 1 | 1 | 0 | 93 | 1 | 0 | 0 | 0 |
| 0 | 14 | 2010 | 41 | -0.86 | 17.8 | 1 | 0 | 0 | 1 | 0 | 0 | 0 | 0 | 109 | 1 | 0 | 0 | 1 |
| 1 | 0 | 2010 | 42 | -0.84 | 17.8 | 0 | 1 | 0 | 0 | 0 | 0 | 0 | 0 | 99 | 1 | 0 | 0 | 1 |
| 0 | 0 | 2010 | 54 | -0.84 | 13.4 | 0 | 0 | 0 | 0 | 1 | 0 | 1 | 0 | 91 | 1 | 0 | 0 | 0 |
| 1 | 18 | 2010 | 43 | -0.84 | 17.8 | 1 | 0 | 0 | 0 | 0 | 1 | 0 | 1 | 99 | 1 | 0 | 0 | 1 |
| 1 | 34 | 2010 | 51 | -0.84 | 13.4 | 0 | 1 | 0 | 0 | 1 | 0 | 0 | 0 | 85 | 1 | 0 | 0 | 0 |
| 0 | 0 | 2010 | 58 | -0.84 | 13.4 | 0 | 0 | 0 | 1 | 0 | 0 | 1 | 0 | 80 | 1 | 0 | 0 | 1 |
| 0 | 0 | 2010 | 51 | -0.83 | 12.5 | 1 | 0 | 0 | 0 | 0 | 1 | 0 | 1 | 90 | 1 | 0 | 0 | 1 |
| 0 | 0 | 2010 | 40 | -0.83 | 21.6 | 0 | 0 | 0 | 0 | 0 | 0 | 0 | 0 | 88 | 1 | 0 | 0 | 1 |
| 0 | 0 | 2010 | 51 | -0.82 | 12.5 | 1 | 0 | 0 | 0 | 1 | 0 | 0 | 0 | 101 | 0 | 0 | 0 | 0 |
| 0 | 0 | 2010 | 40 | -0.82 | 21.6 | 0 | 0 | 0 | 1 | 0 | 0 | 0 | 0 | 85 | 1 | 0 | 0 | 0 |
| 0 | 2 | 2010 | 50 | -0.82 | 12.5 | 1 | 0 | 0 | 0 | 0 | 1 | 0 | 1 | 110 | 1 | 0 | 0 | 1 |
| 0 | 0 | 2010 | 52 | -0.77 | 12.4 | 0 | 0 | 1 | 0 | 0 | 0 | 0 | 0 | 94 | 1 | 0 | 0 | 0 |
| 0 | 6 | 2010 | 52 | -0.77 | 12.4 | 1 | 0 | 0 | 0 | 0 | 1 | 0 | 1 | 98 | 1 | 0 | 0 | 0 |
| 0 | 0 | 2010 | 53 | -0.75 | 12.4 | 0 | 0 | 1 | 0 | 0 | 0 | 0 | 0 | 100 | 0 | 0 | 0 | 0 |
| 0 | 0 | 2010 | 53 | -0.75 | 12.4 | 1 | 0 | 0 | 0 | 0 | 1 | 0 | 1 | 105 | 0 | 0 | 0 | 1 |
| 1 | 28 | 2010 | 42 | -0.60 | 23.2 | 0 | 0 | 0 | 0 | 0 | 0 | 0 | 0 | 116 | 1 | 0 | 0 | 1 |
| 1 | 0 | 2010 | 51 | -0.60 | 18.9 | 0 | 1 | 0 | 0 | 0 | 1 | 0 | 0 | 111 | 1 | 0 | 1 | 1 |
| 1 | 2 | 2010 | 51 | -0.59 | 18.9 | 1 | 0 | 0 | 0 | 0 | 1 | 0 | 0 | 105 | 0 | 1 | 1 | 1 |
| 0 | 0 | 2010 | 51 | -0.59 | 18.9 | 1 | 0 | 0 | 0 | 0 | 1 | 0 | 0 | 106 | 1 | 0 | 0 | 1 |
| 0 | 9 | 2010 | 26 | -0.59 | 26.0 | 0 | 1 | 0 | 0 | 0 | 1 | 1 | 0 | 86 | 0 | 1 | 0 | 1 |
| 1 | 10 | 2010 | 24 | -0.59 | 26.0 | 0 | 1 | 0 | 0 | 0 | 1 | 1 | 0 | 100 | 0 | 1 | 0 | 1 |
| 0 | 20 | 2010 | 26 | -0.59 | 25.7 | 1 | 0 | 0 | 1 | 0 | 0 | 0 | 0 | 89 | 0 | 0 | 0 | 1 |
| 1 | 38 | 2010 | 28 | -0.59 | 25.7 | 0 | 1 | 0 | 0 | 0 | 1 | 0 | 1 | 91 | 1 | 0 | 0 | 1 |
| 1 | 1 | 2010 | 51 | -0.59 | 18.9 | 1 | 0 | 0 | 0 | 0 | 0 | 0 | 0 | 102 | 0 | 0 | 0 | 1 |
| 0 | 9 | 2010 | 30 | -0.59 | 25.7 | 1 | 0 | 0 | 0 | 1 | 0 | 1 | 0 | 104 | 1 | 0 | 0 | 1 |
| 1 | 39 | 2010 | 26 | -0.59 | 25.7 | 0 | 1 | 0 | 1 | 0 | 0 | 0 | 0 | 95 | 0 | 0 | 0 | 0 |
| 0 | 12 | 2010 | 26 | -0.58 | 25.7 | 0 | 1 | 0 | 0 | 1 | 0 | 0 | 0 | 100 | 1 | 0 | 0 | 1 |
| 0 | 0 | 2010 | 26 | -0.58 | 19.7 | 0 | 1 | 0 | 1 | 0 | 0 | 0 | 0 | 92 | 0 | 0 | 0 | 1 |
| 0 | 0 | 2010 | 23 | -0.58 | 19.7 | 0 | 0 | 1 | 0 | 0 | 1 | 0 | 1 | 95 | 1 | 0 | 0 | 0 |
| 0 | 1 | 2010 | 23 | -0.58 | 19.7 | 0 | 1 | 0 | 0 | 0 | 1 | 0 | 1 | 98 | 0 | 1 | 0 | 1 |
| 1 | 12 | 2010 | 25 | -0.58 | 19.7 | 0 | 1 | 0 | 0 | 1 | 0 | 1 | 0 | 93 | 1 | 0 | 0 | 1 |
| 0 | 0 | 2010 | 26 | -0.58 | 19.7 | 0 | 0 | 1 | 0 | 0 | 1 | 1 | 0 | 103 | 1 | 0 | 0 | 1 |
| 0 | 0 | 2010 | 30 | -0.47 | 26.8 | 1 | 0 | 0 | 0 | 1 | 0 | 0 | 0 | 90 | 1 | 0 | 0 | 0 |
| 0 | 3 | 2010 | 34 | -0.47 | 26.8 | 1 | 0 | 0 | 1 | 0 | 0 | 0 | 0 | 109 | 0 | 1 | 0 | 0 |
| 0 | 8 | 2010 | 30 | -0.47 | 26.8 | 0 | 0 | 0 | 1 | 0 | 0 | 1 | 0 | 104 | 1 | 0 | 0 | 0 |
| 0 | 0 | 2010 | 32 | -0.46 | 26.8 | 1 | 0 | 0 | 0 | 0 | 0 | 0 | 0 | 118 | 1 | 0 | 0 | 0 |
| 0 | 0 | 2010 | 34 | -0.13 | 27.1 | 0 | 0 | 0 | 0 | 0 | 0 | 0 | 0 | 97 | 0 | 0 | 0 | 0 |
| 0 | 0 | 2010 | 34 | -0.13 | 27.1 | 0 | 0 | 0 | 0 | 0 | 0 | 0 | 0 | 116 | 0 | 1 | 0 | 0 |
| 0 | 1 | 2010 | 34 | -0.13 | 27.1 | 0 | 0 | 0 | 0 | 0 | 0 | 0 | 0 | 103 | 0 | 0 | 0 | 0 |
| 0 | 19 | 2010 | 36 | -0.11 | 27.1 | 0 | 0 | 1 | 0 | 1 | 0 | 0 | 0 | 129 | 0 | 1 | 0 | 0 |
| 0 | 0 | 2010 | 36 | -0.10 | 27.1 | 0 | 0 | 1 | 0 | 0 | 1 | 0 | 1 | 121 | 0 | 0 | 0 | 0 |
| 0 | 0 | 2010 | 36 | -0.10 | 27.2 | 0 | 0 | 0 | 0 | 0 | 0 | 0 | 0 | 131 | 0 | 1 | 0 | 0 |
| 0 | 2 | 2010 | 34 | -0.10 | 27.1 | 1 | 0 | 0 | 1 | 0 | 0 | 0 | 0 | 110 | 1 | 0 | 0 | 0 |
| 0 | 1 | 2010 | 34 | -0.10 | 27.1 | 1 | 0 | 0 | 0 | 1 | 0 | 1 | 0 | 104 | 0 | 0 | 0 | 0 |
| 1 | 41 | 2010 | 36 | -0.10 | 27.2 | 1 | 0 | 0 | 1 | 0 | 0 | 0 | 0 | 125 | 0 | 1 | 0 | 0 |
| 0 | 19 | 2010 | 34 | -0.10 | 27.1 | 1 | 0 | 0 | 0 | 0 | 1 | 1 | 0 | 91 | 0 | 1 | 0 | 0 |
| 1 | 13 | 2010 | 36 | -0.10 | 27.2 | 1 | 0 | 0 | 1 | 0 | 0 | 0 | 0 | 119 | 1 | 0 | 0 | 0 |
| 1 | 16 | 2010 | 34 | -0.09 | 27.2 | 1 | 0 | 0 | 1 | 0 | 0 | 0 | 0 | 89 | 1 | 0 | 0 | 0 |
| 0 | 1 | 2010 | 36 | -0.07 | 27.0 | 1 | 0 | 0 | 1 | 0 | 0 | 1 | 0 | 122 | 1 | 0 | 1 | 0 |
| 0 | 8 | 2010 | 36 | -0.07 | 27.0 | 1 | 0 | 0 | 1 | 0 | 0 | 0 | 0 | 111 | 1 | 0 | 1 | 0 |
| 0 | 0 | 2010 | 36 | -0.07 | 27.0 | 1 | 0 | 0 | 1 | 0 | 0 | 0 | 0 | 104 | 1 | 0 | 0 | 0 |
| 1 | 28 | 2010 | 34 | -0.07 | 27.0 | 1 | 0 | 0 | 1 | 0 | 0 | 0 | 0 | 90 | 1 | 0 | 0 | 0 |
| 0 | 23 | 2010 | 36 | -0.07 | 27.0 | 1 | 0 | 0 | 1 | 0 | 0 | 0 | 0 | 82 | 1 | 0 | 0 | 0 |
| 0 | 0 | 2010 | 42 | -0.07 | 27.3 | 1 | 0 | 0 | 0 | 0 | 0 | 0 | 0 | 87 | 1 | 0 | 0 | 0 |
| 0 | 0 | 2010 | 40 | -0.06 | 27.3 | 1 | 0 | 0 | 0 | 0 | 0 | 0 | 0 | 99 | 0 | 0 | 0 | 0 |
| 0 | 6 | 2010 | 42 | -0.06 | 27.3 | 1 | 0 | 0 | 1 | 0 | 0 | 0 | 0 | 133 | 0 | 1 | 0 | 0 |
| 0 | 11 | 2010 | 36 | 0.00 | 20.3 | 0 | 1 | 0 | 0 | 0 | 1 | 0 | 0 | 73 | 1 | 0 | 0 | 0 |
| 1 | 13 | 2010 | 54 | 0.00 | 17.0 | 0 | 0 | 1 | 0 | 0 | 1 | 1 | 0 | 94 | 0 | 1 | 0 | 0 |
| 0 | 0 | 2010 | 36 | 0.00 | 20.3 | 1 | 0 | 0 | 1 | 0 | 0 | 0 | 0 | 85 | 1 | 0 | 0 | 0 |
| 0 | 0 | 2010 | 54 | 0.00 | 17.0 | 0 | 0 | 1 | 0 | 0 | 1 | 0 | 1 | 88 | 0 | 1 | 0 | 0 |
| 0 | 0 | 2010 | 35 | 0.00 | 20.3 | 1 | 0 | 0 | 1 | 0 | 0 | 0 | 0 | 92 | 0 | 0 | 0 | 0 |
| 0 | 0 | 2010 | 35 | 0.01 | 20.3 | 0 | 1 | 0 | 0 | 0 | 1 | 0 | 1 | 98 | 1 | 0 | 0 | 0 |
| 0 | 5 | 2010 | 37 | 0.01 | 20.3 | 0 | 1 | 0 | 1 | 0 | 0 | 0 | 0 | 95 | 1 | 0 | 0 | 0 |
| 0 | 0 | 2010 | 54 | 0.01 | 17.0 | 0 | 1 | 0 | 1 | 0 | 0 | 0 | 0 | 90 | 1 | 0 | 0 | 0 |
| 0 | 0 | 2010 | 52 | 0.01 | 17.0 | 0 | 1 | 0 | 0 | 0 | 0 | 0 | 0 | 90 | 1 | 0 | 0 | 0 |
| 0 | 0 | 2010 | 54 | 0.02 | 17.0 | 0 | 1 | 0 | 0 | 1 | 0 | 0 | 1 | 94 | 0 | 0 | 0 | 0 |
| 0 | 2 | 2010 | 54 | 0.06 | 26.1 | 0 | 1 | 0 | 0 | 0 | 1 | 0 | 1 | 119 | 0 | 0 | 0 | 0 |
| 0 | 14 | 2010 | 53 | 0.07 | 26.1 | 0 | 0 | 1 | 0 | 1 | 0 | 0 | 0 | 118 | 1 | 0 | 0 | 0 |
| 0 | 0 | 2010 | 53 | 0.07 | 26.1 | 0 | 1 | 0 | 1 | 0 | 0 | 0 | 0 | 109 | 0 | 0 | 0 | 0 |
| 0 | 0 | 2010 | 53 | 0.07 | 26.1 | 0 | 1 | 0 | 0 | 0 | 1 | 1 | 0 | 102 | 0 | 0 | 0 | 0 |
| 1 | 41 | 2010 | 42 | 0.08 | 25.3 | 0 | 0 | 0 | 0 | 0 | 0 | 0 | 0 | 104 | 0 | 1 | 0 | 0 |
| 0 | 0 | 2010 | 42 | 0.08 | 25.3 | 1 | 0 | 0 | 0 | 0 | 0 | 0 | 0 | 117 | 1 | 0 | 0 | 0 |
| 1 | 0 | 2010 | 55 | 0.08 | 26.1 | 0 | 1 | 0 | 1 | 0 | 0 | 1 | 0 | 89 | 1 | 0 | 0 | 0 |
| 0 | 0 | 2010 | 41 | 0.08 | 25.3 | 1 | 0 | 0 | 0 | 1 | 0 | 0 | 0 | 141 | 1 | 0 | 0 | 0 |
| 0 | 0 | 2010 | 52 | 0.10 | 24.0 | 0 | 1 | 0 | 0 | 0 | 1 | 1 | 0 | 123 | 0 | 1 | 0 | 0 |
| 0 | 0 | 2010 | 56 | 0.11 | 24.0 | 0 | 1 | 0 | 0 | 0 | 1 | 1 | 0 | 112 | 1 | 0 | 0 | 0 |
| 0 | 1 | 2010 | 56 | 0.11 | 24.0 | 1 | 0 | 0 | 1 | 0 | 0 | 0 | 0 | 81 | 0 | 1 | 0 | 0 |
| 0 | 0 | 2010 | 54 | 0.13 | 19.2 | 0 | 1 | 0 | 0 | 0 | 1 | 0 | 1 | 106 | 1 | 0 | 0 | 0 |
| 0 | 0 | 2010 | 54 | 0.13 | 19.2 | 0 | 1 | 0 | 0 | 0 | 1 | 1 | 0 | 104 | 0 | 1 | 0 | 0 |
| 0 | 0 | 2010 | 54 | 0.14 | 19.2 | 0 | 1 | 0 | 0 | 0 | 1 | 1 | 0 | 102 | 1 | 0 | 0 | 0 |
| 0 | 0 | 2010 | 53 | 0.15 | 19.8 | 1 | 0 | 0 | 0 | 0 | 1 | 0 | 0 | 112 | 0 | 0 | 0 | 1 |
| 0 | 0 | 2010 | 53 | 0.16 | 19.8 | 1 | 0 | 0 | 0 | 0 | 1 | 0 | 0 | 114 | 1 | 0 | 0 | 0 |
| 0 | 0 | 2010 | 55 | 0.17 | 19.8 | 0 | 1 | 0 | 0 | 0 | 1 | 1 | 0 | 108 | 1 | 0 | 0 | 0 |
| 0 | 0 | 2010 | 57 | 0.17 | 19.8 | 0 | 1 | 0 | 0 | 0 | 1 | 0 | 1 | 103 | 0 | 1 | 0 | 0 |
| 0 | 0 | 2010 | 53 | 0.17 | 19.8 | 0 | 1 | 0 | 0 | 0 | 1 | 0 | 1 | 106 | 0 | 0 | 0 | 0 |
| 0 | 0 | 2010 | 52 | 0.18 | 20.8 | 1 | 0 | 0 | 0 | 1 | 0 | 1 | 0 | 93 | 1 | 0 | 0 | 0 |
| 0 | 0 | 2010 | 54 | 0.19 | 20.8 | 0 | 0 | 1 | 0 | 0 | 1 | 0 | 0 | 93 | 0 | 1 | 0 | 0 |
| 0 | 0 | 2010 | 51 | 0.19 | 20.8 | 0 | 0 | 1 | 0 | 0 | 1 | 0 | 1 | 91 | 0 | 0 | 0 | 0 |
| 0 | 0 | 2010 | 50 | 0.20 | 20.8 | 0 | 1 | 0 | 0 | 0 | 1 | 0 | 1 | 88 | 1 | 0 | 0 | 0 |
| 0 | 0 | 2010 | 50 | 0.20 | 20.8 | 0 | 1 | 0 | 0 | 0 | 1 | 0 | 0 | 89 | 0 | 1 | 0 | 0 |
| 0 | 0 | 2010 | 64 | 0.46 | 26.3 | 0 | 1 | 0 | 0 | 0 | 1 | 1 | 0 | 135 | 0 | 1 | 0 | 1 |
| 0 | 0 | 2010 | 60 | 0.49 | 18.0 | 0 | 0 | 1 | 0 | 0 | 1 | 1 | 0 | 117 | 1 | 0 | 0 | 0 |
| 0 | 0 | 2010 | 50 | 0.49 | 18.0 | 0 | 1 | 0 | 0 | 1 | 0 | 0 | 0 | 119 | 1 | 0 | 0 | 0 |
| 0 | 0 | 2010 | 51 | 0.50 | 18.0 | 0 | 1 | 0 | 0 | 0 | 1 | 1 | 0 | 118 | 1 | 0 | 0 | 0 |
| 0 | 0 | 2010 | 51 | 0.51 | 18.0 | 0 | 0 | 1 | 0 | 0 | 1 | 0 | 0 | 105 | 0 | 1 | 0 | 0 |
| 0 | 0 | 2010 | 57 | 0.51 | 17.6 | 0 | 1 | 0 | 0 | 0 | 1 | 0 | 0 | 100 | 1 | 0 | 0 | 0 |
| 0 | 0 | 2010 | 53 | 0.52 | 17.6 | 1 | 0 | 0 | 0 | 0 | 1 | 0 | 0 | 100 | 1 | 0 | 0 | 0 |
| 0 | 0 | 2010 | 38 | 0.57 | 29.1 | 0 | 0 | 0 | 0 | 0 | 0 | 0 | 0 | 91 | 0 | 0 | 0 | 1 |
| 0 | 0 | 2010 | 36 | 0.57 | 29.1 | 0 | 1 | 0 | 0 | 1 | 0 | 0 | 0 | 106 | 0 | 1 | 0 | 1 |
| 0 | 0 | 2010 | 38 | 0.57 | 29.1 | 0 | 0 | 0 | 0 | 0 | 0 | 0 | 0 | 97 | 0 | 0 | 0 | 1 |
| 0 | 0 | 2010 | 50 | 0.61 | 27.4 | 0 | 0 | 0 | 0 | 1 | 0 | 0 | 0 | 121 | 1 | 0 | 0 | 1 |
| 0 | 0 | 2010 | 52 | 0.62 | 27.4 | 0 | 0 | 0 | 0 | 0 | 0 | 0 | 0 | 114 | 0 | 1 | 0 | 0 |
| 0 | 0 | 2010 | 50 | 0.62 | 27.4 | 0 | 0 | 0 | 0 | 0 | 0 | 0 | 0 | 105 | 0 | 0 | 0 | 0 |
| 0 | 0 | 2010 | 52 | 0.62 | 27.4 | 1 | 0 | 0 | 0 | 1 | 0 | 0 | 0 | 83 | 1 | 0 | 0 | 0 |
| 0 | 0 | 2010 | 50 | 0.63 | 27.4 | 1 | 0 | 0 | 0 | 1 | 0 | 0 | 0 | 91 | 0 | 0 | 0 | 0 |
| 0 | 0 | 2010 | 52 | 0.63 | 26.8 | 0 | 0 | 0 | 0 | 0 | 0 | 0 | 0 | 89 | 1 | 0 | 0 | 0 |
| 0 | 0 | 2010 | 58 | 0.63 | 26.8 | 1 | 0 | 0 | 1 | 0 | 0 | 0 | 0 | 101 | 1 | 0 | 0 | 1 |
| 0 | 0 | 2010 | 44 | 0.78 | 24.9 | 0 | 0 | 0 | 0 | 0 | 0 | 0 | 0 | 116 | 1 | 0 | 0 | 1 |
| 0 | 0 | 2010 | 44 | 0.78 | 24.9 | 0 | 0 | 0 | 1 | 0 | 0 | 0 | 0 | 95 | 1 | 0 | 0 | 1 |
| 0 | 0 | 2010 | 44 | 0.78 | 24.9 | 1 | 0 | 0 | 0 | 0 | 0 | 0 | 0 | 107 | 0 | 0 | 0 | 1 |
| 1 | 0 | 2010 | 32 | 0.89 | 22.1 | 1 | 0 | 0 | 0 | 1 | 0 | 0 | 0 | 105 | 1 | 0 | 0 | 0 |
| 0 | 0 | 2010 | 33 | 0.89 | 22.1 | 0 | 0 | 0 | 0 | 0 | 0 | 0 | 0 | 110 | 1 | 0 | 0 | 0 |
| 0 | 0 | 2010 | 34 | 0.90 | 21.9 | 1 | 0 | 0 | 1 | 0 | 0 | 0 | 0 | 106 | 0 | 0 | 0 | 0 |
| 0 | 20 | 2010 | 33 | 0.90 | 21.9 | 1 | 0 | 0 | 0 | 1 | 0 | 0 | 0 | 110 | 1 | 0 | 0 | 0 |
| 0 | 0 | 2010 | 33 | 0.91 | 22.5 | 1 | 0 | 0 | 0 | 0 | 0 | 0 | 0 | 92 | 1 | 0 | 0 | 0 |
| 1 | 0 | 2010 | 34 | 0.91 | 22.5 | 0 | 1 | 0 | 0 | 0 | 1 | 0 | 0 | 96 | 1 | 0 | 0 | 0 |
| 0 | 0 | 2010 | 33 | 0.91 | 22.5 | 0 | 1 | 0 | 0 | 1 | 0 | 0 | 0 | 103 | 0 | 0 | 0 | 0 |
| 0 | 0 | 2010 | 33 | 0.91 | 22.5 | 0 | 0 | 0 | 1 | 0 | 0 | 0 | 0 | 98 | 1 | 0 | 0 | 0 |
| 0 | 0 | 2010 | 48 | 1.18 | 20.6 | 0 | 1 | 0 | 0 | 0 | 1 | 1 | 0 | 106 | 1 | 0 | 0 | 0 |
| 0 | 0 | 2010 | 49 | 1.18 | 20.6 | 0 | 1 | 0 | 0 | 0 | 1 | 0 | 1 | 110 | 0 | 1 | 0 | 1 |
| 0 | 0 | 2010 | 47 | 1.18 | 20.6 | 0 | 0 | 1 | 0 | 0 | 1 | 0 | 1 | 118 | 1 | 0 | 0 | 1 |
| 0 | 0 | 2010 | 49 | 1.19 | 20.6 | 0 | 0 | 1 | 1 | 0 | 0 | 1 | 0 | 118 | 0 | 1 | 0 | 1 |
| 0 | 20 | 2010 | 37 | 1.35 | 19.3 | 0 | 0 | 0 | 0 | 0 | 0 | 0 | 0 | 107 | 0 | 0 | 0 | 1 |
| 0 | 0 | 2010 | 36 | 1.35 | 19.3 | 0 | 0 | 0 | 0 | 0 | 0 | 0 | 0 | 106 | 0 | 1 | 0 | 1 |
| 0 | 0 | 2010 | 37 | 1.36 | 19.3 | 1 | 0 | 0 | 0 | 0 | 0 | 0 | 0 | 111 | 1 | 0 | 0 | 1 |
| 0 | 0 | 2010 | 30 | 1.47 | 27.2 | 1 | 0 | 0 | 0 | 0 | 0 | 0 | 0 | 113 | 1 | 0 | 1 | 1 |
| 0 | 0 | 2010 | 32 | 1.48 | 27.2 | 0 | 0 | 0 | 0 | 0 | 0 | 0 | 0 | 104 | 0 | 1 | 0 | 1 |
| 0 | 0 | 2010 | 30 | 1.48 | 27.2 | 0 | 0 | 0 | 0 | 0 | 0 | 0 | 0 | 126 | 0 | 0 | 0 | 1 |
| 0 | 0 | 2010 | 24 | 1.52 | 28.8 | 1 | 0 | 0 | 1 | 0 | 0 | 0 | 0 | 79 | 0 | 0 | 0 | 1 |
| 0 | 0 | 2010 | 33 | 1.61 | 24.2 | 0 | 1 | 0 | 0 | 0 | 1 | 0 | 0 | 104 | 1 | 0 | 0 | 0 |
| 0 | 0 | 2010 | 31 | 1.61 | 23.6 | 1 | 0 | 0 | 1 | 0 | 0 | 0 | 0 | 104 | 0 | 1 | 0 | 1 |
| 1 | 12 | 2010 | 31 | 1.61 | 23.6 | 1 | 0 | 0 | 0 | 1 | 0 | 0 | 0 | 98 | 0 | 0 | 0 | 0 |
| 1 | 5 | 2010 | 30 | 1.61 | 23.6 | 1 | 0 | 0 | 0 | 1 | 0 | 0 | 0 | 98 | 1 | 0 | 0 | 1 |
| 0 | 0 | 2010 | 33 | 1.61 | 24.2 | 1 | 0 | 0 | 0 | 0 | 0 | 0 | 0 | 116 | 1 | 0 | 0 | 0 |
| 0 | 5 | 2010 | 32 | 1.61 | 24.2 | 0 | 1 | 0 | 0 | 1 | 0 | 1 | 0 | 111 | 1 | 0 | 0 | 0 |
| 0 | 0 | 2010 | 32 | 1.61 | 24.2 | 1 | 0 | 0 | 0 | 0 | 0 | 0 | 0 | 119 | 0 | 0 | 0 | 0 |
| 1 | 25 | 2010 | 32 | 1.62 | 24.2 | 0 | 1 | 0 | 0 | 1 | 0 | 0 | 0 | 117 | 1 | 0 | 0 | 0 |
| 0 | 0 | 2011 | 24 | -2.25 | 16.3 | 0 | 0 | 1 | 1 | 0 | 0 | 0 | 0 | 107 | 1 | 0 | 0 | 0 |
| 0 | 0 | 2011 | 23 | -2.24 | 16.3 | 0 | 1 | 0 | 0 | 1 | 0 | 0 | 0 | 106 | 0 | 0 | 0 | 0 |
| 0 | 0 | 2011 | 23 | -2.24 | 16.3 | 0 | 0 | 1 | 0 | 1 | 0 | 1 | 0 | 98 | 0 | 1 | 0 | 0 |
| 0 | 1 | 2011 | 44 | -2.22 | 23.4 | 0 | 1 | 0 | 0 | 0 | 0 | 0 | 0 | 104 | 1 | 0 | 0 | 1 |
| 0 | 0 | 2011 | 43 | -2.21 | 23.4 | 1 | 0 | 0 | 0 | 1 | 0 | 0 | 0 | 108 | 1 | 0 | 0 | 1 |
| 0 | 0 | 2011 | 44 | -2.21 | 23.4 | 0 | 1 | 0 | 1 | 0 | 0 | 0 | 0 | 111 | 0 | 0 | 0 | 1 |
| 0 | 0 | 2011 | 45 | -2.21 | 24.2 | 1 | 0 | 0 | 1 | 0 | 0 | 0 | 0 | 90 | 0 | 1 | 0 | 1 |
| 0 | 0 | 2011 | 45 | -2.21 | 24.2 | 1 | 0 | 0 | 1 | 0 | 0 | 0 | 0 | 93 | 1 | 0 | 0 | 1 |
| 0 | 0 | 2011 | 46 | -2.21 | 23.4 | 1 | 0 | 0 | 1 | 0 | 0 | 0 | 0 | 110 | 0 | 0 | 0 | 1 |
| 0 | 0 | 2011 | 43 | -2.21 | 24.2 | 1 | 0 | 0 | 1 | 0 | 0 | 0 | 0 | 100 | 0 | 0 | 0 | 1 |
| 0 | 0 | 2011 | 43 | -2.20 | 24.2 | 0 | 1 | 0 | 0 | 1 | 0 | 0 | 0 | 104 | 0 | 1 | 0 | 1 |
| 0 | 0 | 2011 | 47 | -2.20 | 23.4 | 1 | 0 | 0 | 0 | 0 | 0 | 0 | 0 | 111 | 1 | 0 | 0 | 1 |
| 0 | 0 | 2011 | 44 | -2.20 | 24.2 | 1 | 0 | 0 | 0 | 1 | 0 | 1 | 0 | 99 | 0 | 1 | 0 | 1 |
| 0 | 0 | 2011 | 49 | -2.20 | 23.4 | 1 | 0 | 0 | 0 | 0 | 0 | 0 | 0 | 113 | 1 | 0 | 0 | 1 |
| 0 | 0 | 2011 | 24 | -2.16 | 15.8 | 0 | 1 | 0 | 1 | 0 | 0 | 0 | 0 | 98 | 0 | 0 | 0 | 0 |
| 0 | 0 | 2011 | 24 | -2.16 | 15.8 | 1 | 0 | 0 | 1 | 0 | 0 | 0 | 0 | 99 | 0 | 0 | 0 | 1 |
| 0 | 0 | 2011 | 24 | -2.16 | 15.8 | 0 | 1 | 0 | 0 | 0 | 1 | 0 | 1 | 96 | 0 | 1 | 0 | 1 |
| 0 | 0 | 2011 | 24 | -2.15 | 15.8 | 0 | 1 | 0 | 0 | 1 | 0 | 0 | 0 | 95 | 0 | 0 | 0 | 1 |
| 0 | 0 | 2011 | 25 | -2.15 | 15.8 | 0 | 1 | 0 | 0 | 0 | 1 | 0 | 0 | 96 | 0 | 0 | 0 | 0 |
| 0 | 0 | 2011 | 26 | -2.15 | 15.8 | 0 | 1 | 0 | 0 | 1 | 0 | 0 | 0 | 88 | 1 | 0 | 0 | 1 |
| 0 | 0 | 2011 | 24 | -2.07 | 16.9 | 0 | 0 | 1 | 0 | 0 | 1 | 1 | 0 | 97 | 1 | 0 | 0 | 0 |
| 0 | 0 | 2011 | 26 | -2.07 | 22.4 | 0 | 0 | 0 | 0 | 1 | 0 | 1 | 0 | 138 | 0 | 0 | 0 | 1 |
| 0 | 0 | 2011 | 24 | -2.06 | 16.9 | 0 | 1 | 0 | 0 | 1 | 0 | 0 | 0 | 94 | 1 | 0 | 0 | 0 |
| 0 | 0 | 2011 | 25 | -2.06 | 22.4 | 0 | 0 | 0 | 1 | 0 | 0 | 0 | 0 | 153 | 0 | 0 | 0 | 1 |
| 0 | 0 | 2011 | 25 | -2.06 | 16.9 | 1 | 0 | 0 | 0 | 1 | 0 | 1 | 0 | 92 | 1 | 0 | 0 | 0 |
| 0 | 0 | 2011 | 27 | -2.05 | 22.4 | 1 | 0 | 0 | 1 | 0 | 0 | 0 | 0 | 140 | 0 | 1 | 0 | 1 |
| 0 | 0 | 2011 | 25 | -2.05 | 16.9 | 0 | 1 | 0 | 1 | 0 | 0 | 1 | 0 | 91 | 0 | 1 | 0 | 0 |
| 0 | 0 | 2011 | 24 | -2.04 | 16.9 | 0 | 0 | 1 | 0 | 1 | 0 | 0 | 0 | 91 | 1 | 0 | 0 | 0 |
| 0 | 4 | 2011 | 25 | -2.04 | 22.4 | 0 | 0 | 0 | 0 | 1 | 0 | 1 | 0 | 154 | 0 | 1 | 0 | 1 |
| 0 | 0 | 2011 | 23 | -2.04 | 16.9 | 1 | 0 | 0 | 0 | 0 | 0 | 0 | 0 | 93 | 0 | 0 | 0 | 0 |
| 1 | 2 | 2011 | 26 | -1.83 | 17.7 | 0 | 1 | 0 | 1 | 0 | 0 | 0 | 0 | 94 | 0 | 1 | 0 | 1 |
| 0 | 0 | 2011 | 24 | -1.83 | 17.7 | 0 | 1 | 0 | 1 | 0 | 0 | 0 | 0 | 92 | 1 | 0 | 1 | 1 |
| 0 | 0 | 2011 | 27 | -1.82 | 17.7 | 1 | 0 | 0 | 1 | 0 | 0 | 0 | 0 | 92 | 0 | 1 | 0 | 1 |
| 0 | 1 | 2011 | 23 | -1.82 | 17.7 | 0 | 1 | 0 | 1 | 0 | 0 | 0 | 0 | 92 | 0 | 0 | 0 | 1 |
| 0 | 0 | 2011 | 24 | -1.81 | 17.7 | 1 | 0 | 0 | 1 | 0 | 0 | 0 | 0 | 93 | 0 | 1 | 0 | 1 |
| 0 | 0 | 2011 | 24 | -1.80 | 17.7 | 0 | 1 | 0 | 0 | 0 | 1 | 1 | 0 | 91 | 0 | 0 | 0 | 1 |
| 1 | 0 | 2011 | 28 | -1.80 | 17.7 | 0 | 1 | 0 | 1 | 0 | 0 | 0 | 0 | 82 | 0 | 0 | 0 | 1 |
| 0 | 0 | 2011 | 52 | -1.80 | 24.7 | 1 | 0 | 0 | 1 | 0 | 0 | 0 | 0 | 111 | 0 | 0 | 0 | 0 |
| 0 | 0 | 2011 | 53 | -1.80 | 24.7 | 0 | 1 | 0 | 0 | 0 | 1 | 0 | 1 | 94 | 0 | 0 | 0 | 1 |
| 1 | 12 | 2011 | 52 | -1.80 | 24.7 | 0 | 0 | 1 | 1 | 0 | 0 | 1 | 0 | 107 | 0 | 1 | 0 | 0 |
| 1 | 0 | 2011 | 57 | -1.79 | 24.7 | 1 | 0 | 0 | 1 | 0 | 0 | 0 | 0 | 91 | 1 | 0 | 0 | 0 |
| 0 | 0 | 2011 | 52 | -1.77 | 24.5 | 0 | 1 | 0 | 0 | 1 | 0 | 1 | 0 | 88 | 1 | 0 | 0 | 0 |
| 0 | 0 | 2011 | 57 | -1.77 | 24.5 | 0 | 1 | 0 | 0 | 0 | 0 | 0 | 0 | 117 | 0 | 1 | 0 | 0 |
| 0 | 0 | 2011 | 54 | -1.77 | 24.5 | 1 | 0 | 0 | 0 | 0 | 0 | 0 | 0 | 106 | 0 | 0 | 0 | 0 |
| 0 | 0 | 2011 | 55 | -1.77 | 24.5 | 1 | 0 | 0 | 1 | 0 | 0 | 0 | 0 | 113 | 0 | 0 | 0 | 0 |
| 0 | 0 | 2011 | 54 | -1.76 | 24.5 | 1 | 0 | 0 | 1 | 0 | 0 | 0 | 0 | 120 | 0 | 1 | 0 | 0 |
| 0 | 0 | 2011 | 57 | -1.76 | 24.5 | 0 | 1 | 0 | 1 | 0 | 0 | 1 | 0 | 124 | 0 | 1 | 0 | 0 |
| 0 | 0 | 2011 | 29 | -1.70 | 16.5 | 0 | 0 | 0 | 0 | 0 | 0 | 0 | 0 | 128 | 1 | 0 | 0 | 1 |
| 0 | 0 | 2011 | 30 | -1.70 | 16.5 | 0 | 0 | 0 | 0 | 0 | 0 | 0 | 0 | 110 | 1 | 0 | 0 | 1 |
| 0 | 0 | 2011 | 29 | -1.69 | 16.5 | 0 | 0 | 0 | 0 | 0 | 0 | 0 | 0 | 96 | 1 | 0 | 0 | 1 |
| 0 | 1 | 2011 | 25 | -1.45 | 24.4 | 0 | 0 | 1 | 0 | 0 | 1 | 1 | 0 | 104 | 1 | 0 | 0 | 1 |
| 1 | 2 | 2011 | 27 | -1.45 | 24.4 | 1 | 0 | 0 | 1 | 0 | 0 | 0 | 0 | 110 | 1 | 0 | 0 | 1 |
| 0 | 0 | 2011 | 54 | -1.15 | 15.4 | 1 | 0 | 0 | 1 | 0 | 0 | 0 | 0 | 100 | 0 | 1 | 0 | 0 |
| 0 | 2 | 2011 | 52 | -1.14 | 15.4 | 0 | 1 | 0 | 1 | 0 | 0 | 0 | 0 | 100 | 0 | 0 | 0 | 0 |
| 1 | 33 | 2011 | 51 | -1.14 | 15.4 | 0 | 1 | 0 | 1 | 0 | 0 | 0 | 0 | 97 | 0 | 1 | 0 | 1 |
| 1 | 1 | 2011 | 51 | -1.14 | 15.4 | 1 | 0 | 0 | 0 | 1 | 0 | 0 | 1 | 97 | 0 | 0 | 0 | 1 |
| 1 | 8 | 2011 | 51 | -1.13 | 15.4 | 1 | 0 | 0 | 0 | 0 | 1 | 0 | 1 | 94 | 0 | 0 | 0 | 1 |
| 1 | 2 | 2011 | 55 | -0.98 | 15.8 | 1 | 0 | 0 | 1 | 0 | 0 | 0 | 0 | 94 | 1 | 0 | 0 | 1 |
| 1 | 3 | 2011 | 54 | -0.97 | 15.8 | 1 | 0 | 0 | 0 | 1 | 0 | 1 | 0 | 93 | 0 | 0 | 0 | 1 |
| 1 | 2 | 2011 | 53 | -0.97 | 15.8 | 0 | 1 | 0 | 0 | 1 | 0 | 1 | 0 | 94 | 0 | 1 | 0 | 0 |
| 1 | 11 | 2011 | 62 | -0.97 | 15.8 | 0 | 1 | 0 | 1 | 0 | 0 | 0 | 0 | 93 | 0 | 0 | 0 | 0 |
| 1 | 10 | 2011 | 53 | -0.97 | 15.5 | 0 | 1 | 0 | 0 | 1 | 0 | 1 | 0 | 95 | 1 | 0 | 0 | 0 |
| 1 | 7 | 2011 | 52 | -0.97 | 15.5 | 0 | 0 | 1 | 1 | 0 | 0 | 0 | 0 | 96 | 0 | 1 | 0 | 0 |
| 0 | 8 | 2011 | 52 | -0.96 | 15.5 | 0 | 0 | 1 | 1 | 0 | 0 | 0 | 0 | 96 | 0 | 0 | 0 | 0 |
| 1 | 19 | 2011 | 53 | -0.96 | 15.5 | 0 | 1 | 0 | 0 | 1 | 0 | 1 | 0 | 97 | 0 | 1 | 0 | 0 |
| 1 | 20 | 2011 | 53 | -0.96 | 15.5 | 0 | 1 | 0 | 0 | 0 | 1 | 1 | 0 | 98 | 0 | 1 | 0 | 0 |
| 1 | 13 | 2011 | 54 | -0.96 | 15.5 | 0 | 1 | 0 | 0 | 0 | 1 | 1 | 0 | 92 | 0 | 1 | 0 | 0 |
| 0 | 0 | 2011 | 21 | -0.87 | 21.8 | 0 | 1 | 0 | 1 | 0 | 0 | 0 | 0 | 95 | 1 | 0 | 0 | 1 |
| 0 | 0 | 2011 | 42 | -0.86 | 15.6 | 1 | 0 | 0 | 1 | 0 | 0 | 0 | 0 | 89 | 1 | 0 | 0 | 1 |
| 0 | 0 | 2011 | 43 | -0.85 | 15.6 | 1 | 0 | 0 | 1 | 0 | 0 | 0 | 0 | 91 | 0 | 1 | 0 | 1 |
| 1 | 24 | 2011 | 43 | -0.84 | 15.6 | 0 | 1 | 0 | 0 | 1 | 0 | 1 | 0 | 91 | 0 | 1 | 0 | 1 |
| 0 | 0 | 2011 | 53 | -0.84 | 17.6 | 1 | 0 | 0 | 1 | 0 | 0 | 0 | 0 | 93 | 1 | 0 | 0 | 1 |
| 1 | 2 | 2011 | 53 | -0.84 | 17.6 | 0 | 0 | 1 | 0 | 0 | 1 | 0 | 0 | 91 | 1 | 0 | 0 | 1 |
| 0 | 0 | 2011 | 58 | -0.83 | 17.6 | 1 | 0 | 0 | 0 | 0 | 1 | 1 | 0 | 89 | 0 | 0 | 0 | 1 |
| 0 | 0 | 2011 | 53 | -0.83 | 17.6 | 1 | 0 | 0 | 1 | 0 | 0 | 0 | 0 | 86 | 1 | 0 | 0 | 1 |
| 0 | 0 | 2011 | 40 | -0.83 | 17.2 | 1 | 0 | 0 | 0 | 0 | 0 | 0 | 0 | 91 | 1 | 0 | 0 | 1 |
| 0 | 1 | 2011 | 40 | -0.82 | 17.2 | 0 | 0 | 0 | 1 | 0 | 0 | 0 | 0 | 92 | 1 | 0 | 0 | 1 |
| 0 | 0 | 2011 | 42 | -0.82 | 17.2 | 0 | 0 | 0 | 1 | 0 | 0 | 0 | 0 | 91 | 1 | 0 | 0 | 1 |
| 0 | 0 | 2011 | 54 | -0.82 | 18.0 | 0 | 0 | 0 | 1 | 0 | 0 | 0 | 0 | 100 | 1 | 0 | 0 | 1 |
| 0 | 7 | 2011 | 52 | -0.77 | 15.0 | 1 | 0 | 0 | 1 | 0 | 0 | 1 | 0 | 97 | 1 | 0 | 0 | 0 |
| 0 | 6 | 2011 | 53 | -0.77 | 15.0 | 1 | 0 | 0 | 0 | 0 | 1 | 0 | 0 | 94 | 0 | 1 | 0 | 1 |
| 0 | 0 | 2011 | 52 | -0.77 | 15.0 | 1 | 0 | 0 | 1 | 0 | 0 | 0 | 0 | 93 | 1 | 0 | 0 | 1 |
| 0 | 5 | 2011 | 54 | -0.76 | 15.0 | 0 | 1 | 0 | 1 | 0 | 0 | 0 | 0 | 90 | 0 | 0 | 0 | 1 |
| 0 | 0 | 2011 | 52 | -0.76 | 15.0 | 1 | 0 | 0 | 1 | 0 | 0 | 0 | 0 | 88 | 1 | 0 | 0 | 1 |
| 1 | 0 | 2011 | 52 | -0.76 | 15.0 | 0 | 1 | 0 | 0 | 1 | 0 | 1 | 0 | 93 | 1 | 0 | 0 | 1 |
| 0 | 0 | 2011 | 52 | -0.76 | 15.5 | 0 | 0 | 1 | 0 | 0 | 1 | 0 | 0 | 98 | 0 | 0 | 0 | 1 |
| 0 | 2 | 2011 | 53 | -0.75 | 15.5 | 1 | 0 | 0 | 1 | 0 | 0 | 0 | 0 | 98 | 0 | 1 | 0 | 1 |
| 0 | 0 | 2011 | 52 | -0.75 | 15.5 | 0 | 1 | 0 | 1 | 0 | 0 | 0 | 0 | 97 | 0 | 0 | 0 | 1 |
| 0 | 0 | 2011 | 53 | -0.75 | 15.5 | 0 | 1 | 0 | 0 | 1 | 0 | 0 | 0 | 96 | 0 | 1 | 0 | 1 |
| 0 | 0 | 2011 | 52 | -0.74 | 15.5 | 0 | 1 | 0 | 1 | 0 | 0 | 0 | 0 | 95 | 0 | 1 | 0 | 1 |
| 0 | 1 | 2011 | 43 | -0.73 | 23.6 | 0 | 0 | 0 | 0 | 0 | 0 | 0 | 0 | 101 | 0 | 1 | 0 | 1 |
| 1 | 2 | 2011 | 44 | -0.73 | 23.6 | 1 | 0 | 0 | 1 | 0 | 0 | 0 | 0 | 98 | 0 | 0 | 0 | 1 |
| 1 | 1 | 2011 | 43 | -0.72 | 23.6 | 0 | 0 | 0 | 0 | 0 | 0 | 0 | 0 | 89 | 0 | 1 | 0 | 1 |
| 0 | 0 | 2011 | 54 | -0.72 | 15.0 | 1 | 0 | 0 | 1 | 0 | 0 | 0 | 0 | 98 | 1 | 0 | 0 | 0 |
| 1 | 0 | 2011 | 54 | -0.72 | 15.0 | 0 | 1 | 0 | 1 | 0 | 0 | 1 | 0 | 99 | 0 | 1 | 0 | 0 |
| 0 | 0 | 2011 | 53 | -0.72 | 15.0 | 0 | 1 | 0 | 0 | 1 | 0 | 0 | 1 | 96 | 0 | 1 | 0 | 0 |
| 0 | 1 | 2011 | 53 | -0.72 | 15.0 | 0 | 1 | 0 | 0 | 1 | 0 | 0 | 0 | 95 | 0 | 0 | 0 | 1 |
| 0 | 14 | 2011 | 54 | -0.71 | 15.0 | 0 | 0 | 1 | 0 | 1 | 0 | 0 | 0 | 92 | 0 | 1 | 0 | 1 |
| 0 | 0 | 2011 | 53 | -0.71 | 14.8 | 0 | 1 | 0 | 1 | 0 | 0 | 0 | 0 | 89 | 1 | 0 | 0 | 0 |
| 1 | 1 | 2011 | 53 | -0.71 | 14.8 | 0 | 0 | 1 | 0 | 0 | 1 | 1 | 0 | 90 | 1 | 0 | 0 | 0 |
| 1 | 6 | 2011 | 53 | -0.70 | 14.8 | 0 | 0 | 1 | 0 | 0 | 1 | 1 | 0 | 88 | 0 | 1 | 0 | 1 |
| 0 | 0 | 2011 | 54 | -0.70 | 14.8 | 0 | 1 | 0 | 1 | 0 | 0 | 0 | 0 | 89 | 1 | 0 | 0 | 0 |
| 0 | 0 | 2011 | 56 | -0.70 | 14.8 | 1 | 0 | 0 | 1 | 0 | 0 | 0 | 0 | 90 | 1 | 0 | 0 | 0 |
| 0 | 0 | 2011 | 54 | -0.70 | 14.8 | 1 | 0 | 0 | 1 | 0 | 0 | 0 | 0 | 89 | 0 | 0 | 0 | 0 |
| 0 | 0 | 2011 | 22 | -0.67 | 20.9 | 0 | 0 | 0 | 1 | 0 | 0 | 0 | 0 | 129 | 1 | 0 | 0 | 1 |
| 1 | 13 | 2011 | 22 | -0.67 | 20.9 | 0 | 1 | 0 | 0 | 1 | 0 | 1 | 0 | 144 | 0 | 0 | 0 | 1 |
| 0 | 0 | 2011 | 21 | -0.67 | 20.9 | 0 | 0 | 1 | 0 | 0 | 1 | 0 | 1 | 104 | 1 | 0 | 0 | 1 |
| 0 | 1 | 2011 | 22 | -0.67 | 20.9 | 1 | 0 | 0 | 1 | 0 | 0 | 0 | 0 | 145 | 1 | 0 | 0 | 1 |
| 1 | 22 | 2011 | 24 | -0.65 | 21.2 | 0 | 1 | 0 | 0 | 1 | 0 | 1 | 0 | 90 | 0 | 0 | 0 | 1 |
| 1 | 5 | 2011 | 22 | -0.65 | 21.2 | 1 | 0 | 0 | 1 | 0 | 0 | 0 | 0 | 109 | 1 | 0 | 0 | 1 |
| 0 | 1 | 2011 | 24 | -0.65 | 21.2 | 1 | 0 | 0 | 1 | 0 | 0 | 0 | 0 | 109 | 1 | 0 | 0 | 1 |
| 0 | 12 | 2011 | 25 | -0.65 | 21.2 | 0 | 1 | 0 | 0 | 1 | 0 | 0 | 0 | 110 | 1 | 0 | 0 | 1 |
| 0 | 0 | 2011 | 20 | -0.65 | 21.2 | 0 | 1 | 0 | 0 | 1 | 0 | 0 | 0 | 100 | 1 | 0 | 0 | 1 |
| 0 | 7 | 2011 | 23 | -0.64 | 21.2 | 0 | 1 | 0 | 1 | 0 | 0 | 0 | 0 | 100 | 1 | 0 | 0 | 1 |
| 0 | 3 | 2011 | 31 | -0.62 | 20.0 | 0 | 0 | 0 | 0 | 0 | 0 | 0 | 0 | 130 | 0 | 1 | 0 | 1 |
| 0 | 2 | 2011 | 31 | -0.62 | 20.0 | 1 | 0 | 0 | 1 | 0 | 0 | 0 | 0 | 111 | 0 | 1 | 0 | 1 |
| 0 | 9 | 2011 | 31 | -0.61 | 20.0 | 1 | 0 | 0 | 1 | 0 | 0 | 0 | 0 | 106 | 0 | 1 | 0 | 1 |
| 0 | 3 | 2011 | 29 | -0.61 | 20.0 | 0 | 0 | 1 | 0 | 0 | 1 | 0 | 0 | 94 | 1 | 0 | 0 | 1 |
| 0 | 23 | 2011 | 44 | -0.60 | 20.8 | 1 | 0 | 0 | 1 | 0 | 0 | 0 | 0 | 89 | 1 | 0 | 0 | 1 |
| 0 | 1 | 2011 | 29 | -0.59 | 20.3 | 0 | 0 | 1 | 0 | 0 | 1 | 1 | 0 | 88 | 0 | 0 | 0 | 1 |
| 0 | 8 | 2011 | 29 | -0.59 | 20.3 | 0 | 0 | 1 | 0 | 0 | 1 | 0 | 1 | 107 | 0 | 1 | 0 | 1 |
| 1 | 6 | 2011 | 26 | -0.59 | 24.7 | 0 | 0 | 0 | 0 | 0 | 0 | 0 | 0 | 110 | 1 | 0 | 0 | 1 |
| 0 | 5 | 2011 | 27 | -0.59 | 24.7 | 0 | 0 | 0 | 0 | 0 | 0 | 0 | 0 | 104 | 0 | 1 | 0 | 1 |
| 0 | 0 | 2011 | 32 | -0.59 | 20.3 | 1 | 0 | 0 | 0 | 0 | 0 | 0 | 0 | 99 | 0 | 0 | 0 | 1 |
| 1 | 2 | 2011 | 30 | -0.59 | 20.3 | 0 | 1 | 0 | 0 | 1 | 0 | 1 | 0 | 117 | 1 | 0 | 0 | 1 |
| 1 | 30 | 2011 | 32 | -0.59 | 20.3 | 1 | 0 | 0 | 1 | 0 | 0 | 0 | 0 | 122 | 1 | 0 | 0 | 1 |
| 0 | 1 | 2011 | 28 | -0.58 | 24.7 | 0 | 0 | 0 | 0 | 0 | 0 | 0 | 0 | 88 | 0 | 0 | 0 | 1 |
| 0 | 4 | 2011 | 26 | -0.58 | 21.0 | 0 | 0 | 1 | 0 | 0 | 1 | 0 | 1 | 113 | 1 | 0 | 0 | 1 |
| 1 | 3 | 2011 | 25 | -0.58 | 21.0 | 1 | 0 | 0 | 1 | 0 | 0 | 0 | 0 | 115 | 0 | 0 | 0 | 1 |
| 0 | 6 | 2011 | 27 | -0.58 | 21.0 | 0 | 0 | 1 | 0 | 0 | 1 | 1 | 0 | 121 | 1 | 0 | 0 | 1 |
| 0 | 6 | 2011 | 25 | -0.58 | 21.0 | 0 | 0 | 0 | 0 | 0 | 0 | 0 | 0 | 104 | 1 | 0 | 0 | 1 |
| 0 | 13 | 2011 | 27 | -0.58 | 21.0 | 0 | 0 | 0 | 1 | 0 | 0 | 0 | 0 | 97 | 0 | 1 | 0 | 1 |
| 1 | 7 | 2011 | 24 | -0.58 | 24.7 | 0 | 1 | 0 | 0 | 0 | 1 | 1 | 0 | 84 | 0 | 1 | 0 | 1 |
| 0 | 0 | 2011 | 26 | -0.58 | 21.0 | 0 | 1 | 0 | 1 | 0 | 0 | 0 | 0 | 90 | 0 | 0 | 0 | 1 |
| 0 | 0 | 2011 | 33 | -0.47 | 19.6 | 1 | 0 | 0 | 0 | 0 | 0 | 0 | 0 | 136 | 0 | 0 | 0 | 1 |
| 0 | 0 | 2011 | 31 | -0.47 | 19.6 | 0 | 0 | 0 | 0 | 0 | 0 | 0 | 0 | 127 | 0 | 1 | 0 | 1 |
| 0 | 0 | 2011 | 34 | -0.47 | 19.6 | 0 | 1 | 0 | 0 | 1 | 0 | 0 | 0 | 101 | 1 | 0 | 0 | 1 |
| 0 | 0 | 2011 | 31 | -0.46 | 19.6 | 0 | 0 | 1 | 0 | 1 | 0 | 0 | 0 | 90 | 0 | 1 | 0 | 1 |
| 0 | 5 | 2011 | 33 | -0.45 | 19.4 | 0 | 1 | 0 | 1 | 0 | 0 | 0 | 0 | 104 | 1 | 0 | 0 | 1 |
| 0 | 9 | 2011 | 34 | -0.45 | 20.3 | 0 | 0 | 0 | 1 | 0 | 0 | 0 | 0 | 98 | 0 | 0 | 0 | 1 |
| 1 | 2 | 2011 | 34 | -0.45 | 20.3 | 0 | 1 | 0 | 0 | 1 | 0 | 1 | 0 | 91 | 1 | 0 | 0 | 1 |
| 0 | 0 | 2011 | 33 | -0.45 | 19.4 | 1 | 0 | 0 | 1 | 0 | 0 | 0 | 0 | 124 | 1 | 0 | 0 | 1 |
| 0 | 0 | 2011 | 31 | -0.45 | 19.4 | 1 | 0 | 0 | 0 | 1 | 0 | 1 | 0 | 134 | 1 | 0 | 0 | 1 |
| 1 | 2 | 2011 | 39 | -0.27 | 18.1 | 1 | 0 | 0 | 1 | 0 | 0 | 0 | 0 | 89 | 1 | 0 | 0 | 1 |
| 0 | 0 | 2011 | 40 | -0.27 | 18.1 | 1 | 0 | 0 | 1 | 0 | 0 | 0 | 0 | 94 | 1 | 0 | 0 | 1 |
| 0 | 1 | 2011 | 38 | -0.27 | 18.1 | 1 | 0 | 0 | 1 | 0 | 0 | 0 | 0 | 98 | 0 | 0 | 0 | 1 |
| 0 | 1 | 2011 | 40 | -0.27 | 18.1 | 0 | 0 | 0 | 0 | 0 | 0 | 0 | 0 | 98 | 1 | 0 | 0 | 1 |
| 0 | 2 | 2011 | 43 | -0.27 | 17.5 | 0 | 1 | 0 | 0 | 0 | 1 | 0 | 0 | 90 | 0 | 1 | 0 | 1 |
| 0 | 0 | 2011 | 38 | -0.27 | 18.1 | 0 | 0 | 1 | 1 | 0 | 0 | 0 | 0 | 102 | 0 | 0 | 0 | 1 |
| 0 | 0 | 2011 | 42 | -0.27 | 17.5 | 0 | 1 | 0 | 0 | 0 | 1 | 0 | 0 | 98 | 1 | 0 | 0 | 1 |
| 1 | 0 | 2011 | 43 | -0.26 | 17.5 | 0 | 0 | 1 | 0 | 1 | 0 | 0 | 0 | 97 | 1 | 0 | 0 | 1 |
| 1 | 2 | 2011 | 42 | -0.26 | 17.5 | 0 | 1 | 0 | 1 | 0 | 0 | 0 | 0 | 104 | 0 | 1 | 0 | 1 |
| 1 | 10 | 2011 | 45 | -0.26 | 17.5 | 0 | 0 | 1 | 0 | 1 | 0 | 0 | 0 | 105 | 0 | 1 | 0 | 1 |
| 0 | 0 | 2011 | 43 | -0.26 | 17.5 | 0 | 1 | 0 | 0 | 0 | 1 | 0 | 0 | 114 | 0 | 0 | 0 | 1 |
| 0 | 0 | 2011 | 43 | -0.26 | 17.6 | 0 | 0 | 0 | 0 | 0 | 0 | 0 | 0 | 130 | 1 | 0 | 0 | 1 |
| 0 | 3 | 2011 | 42 | -0.25 | 17.6 | 1 | 0 | 0 | 1 | 0 | 0 | 0 | 0 | 123 | 0 | 1 | 0 | 1 |
| 1 | 9 | 2011 | 44 | -0.25 | 17.6 | 0 | 1 | 0 | 1 | 0 | 0 | 0 | 0 | 120 | 0 | 1 | 0 | 1 |
| 0 | 15 | 2011 | 38 | -0.25 | 17.6 | 0 | 1 | 0 | 1 | 0 | 0 | 0 | 0 | 104 | 0 | 1 | 0 | 1 |
| 0 | 0 | 2011 | 70 | -0.16 | 14.8 | 0 | 1 | 0 | 0 | 1 | 0 | 0 | 0 | 94 | 1 | 0 | 0 | 0 |
| 0 | 1 | 2011 | 67 | -0.16 | 14.8 | 0 | 0 | 1 | 1 | 0 | 0 | 0 | 0 | 101 | 0 | 0 | 0 | 0 |
| 0 | 0 | 2011 | 62 | -0.16 | 14.8 | 1 | 0 | 0 | 1 | 0 | 0 | 0 | 0 | 105 | 1 | 0 | 0 | 0 |
| 0 | 0 | 2011 | 33 | -0.13 | 22.3 | 0 | 1 | 0 | 1 | 0 | 0 | 0 | 0 | 97 | 0 | 0 | 1 | 1 |
| 0 | 0 | 2011 | 34 | -0.13 | 22.3 | 1 | 0 | 0 | 1 | 0 | 0 | 0 | 0 | 101 | 1 | 0 | 0 | 1 |
| 0 | 0 | 2011 | 34 | -0.11 | 17.7 | 0 | 0 | 1 | 0 | 0 | 1 | 0 | 1 | 96 | 0 | 1 | 0 | 0 |
| 1 | 5 | 2011 | 36 | -0.10 | 17.7 | 0 | 0 | 1 | 0 | 0 | 1 | 1 | 0 | 93 | 1 | 0 | 0 | 0 |
| 0 | 5 | 2011 | 36 | -0.10 | 18.0 | 1 | 0 | 0 | 1 | 0 | 0 | 0 | 0 | 94 | 0 | 1 | 0 | 0 |
| 0 | 22 | 2011 | 35 | -0.10 | 17.7 | 0 | 1 | 0 | 0 | 1 | 0 | 0 | 0 | 93 | 1 | 0 | 0 | 0 |
| 0 | 0 | 2011 | 37 | -0.10 | 18.0 | 1 | 0 | 0 | 1 | 0 | 0 | 1 | 0 | 95 | 0 | 0 | 0 | 0 |
| 1 | 10 | 2011 | 36 | -0.10 | 17.7 | 1 | 0 | 0 | 1 | 0 | 0 | 0 | 0 | 91 | 1 | 0 | 0 | 0 |
| 1 | 3 | 2011 | 36 | -0.10 | 18.0 | 1 | 0 | 0 | 1 | 0 | 0 | 1 | 0 | 94 | 1 | 0 | 0 | 0 |
| 0 | 0 | 2011 | 35 | -0.10 | 17.7 | 0 | 1 | 0 | 0 | 1 | 0 | 1 | 0 | 90 | 0 | 0 | 0 | 0 |
| 0 | 9 | 2011 | 36 | -0.10 | 18.0 | 1 | 0 | 0 | 1 | 0 | 0 | 1 | 0 | 93 | 0 | 0 | 0 | 0 |
| 0 | 2 | 2011 | 36 | -0.10 | 17.7 | 0 | 1 | 0 | 0 | 1 | 0 | 1 | 0 | 88 | 0 | 0 | 0 | 0 |
| 1 | 19 | 2011 | 34 | -0.10 | 18.0 | 0 | 1 | 0 | 0 | 1 | 0 | 0 | 1 | 92 | 0 | 1 | 0 | 0 |
| 0 | 14 | 2011 | 36 | -0.09 | 18.0 | 0 | 1 | 0 | 1 | 0 | 0 | 0 | 0 | 90 | 1 | 0 | 0 | 0 |
| 0 | 0 | 2011 | 34 | -0.08 | 21.6 | 1 | 0 | 0 | 1 | 0 | 0 | 1 | 0 | 92 | 0 | 0 | 0 | 1 |
| 0 | 0 | 2011 | 34 | -0.08 | 21.6 | 0 | 1 | 0 | 1 | 0 | 0 | 0 | 0 | 90 | 0 | 1 | 0 | 1 |
| 0 | 0 | 2011 | 36 | -0.08 | 21.6 | 0 | 0 | 1 | 0 | 0 | 1 | 1 | 0 | 90 | 1 | 0 | 0 | 1 |
| 0 | 0 | 2011 | 34 | -0.07 | 21.6 | 0 | 0 | 1 | 0 | 0 | 1 | 0 | 0 | 89 | 0 | 1 | 0 | 1 |
| 0 | 5 | 2011 | 37 | 0.00 | 21.8 | 0 | 1 | 0 | 0 | 1 | 0 | 0 | 0 | 101 | 0 | 1 | 0 | 1 |
| 0 | 0 | 2011 | 53 | 0.00 | 17.2 | 0 | 1 | 0 | 0 | 1 | 0 | 0 | 0 | 87 | 1 | 0 | 0 | 1 |
| 0 | 0 | 2011 | 38 | 0.00 | 21.8 | 1 | 0 | 0 | 1 | 0 | 0 | 0 | 0 | 100 | 1 | 0 | 0 | 0 |
| 0 | 3 | 2011 | 53 | 0.00 | 17.2 | 0 | 1 | 0 | 1 | 0 | 0 | 0 | 0 | 88 | 0 | 1 | 0 | 1 |
| 0 | 2 | 2011 | 37 | 0.00 | 21.8 | 0 | 1 | 0 | 0 | 1 | 0 | 0 | 0 | 98 | 1 | 0 | 0 | 0 |
| 0 | 0 | 2011 | 53 | 0.01 | 17.2 | 0 | 0 | 1 | 0 | 1 | 0 | 0 | 0 | 90 | 0 | 0 | 0 | 1 |
| 0 | 6 | 2011 | 38 | 0.01 | 21.8 | 0 | 1 | 0 | 0 | 1 | 0 | 0 | 1 | 91 | 1 | 0 | 0 | 1 |
| 1 | 3 | 2011 | 53 | 0.01 | 17.2 | 0 | 1 | 0 | 0 | 1 | 0 | 0 | 0 | 92 | 1 | 0 | 0 | 1 |
| 0 | 6 | 2011 | 51 | 0.01 | 17.2 | 0 | 1 | 0 | 0 | 0 | 1 | 0 | 1 | 94 | 1 | 0 | 0 | 1 |
| 0 | 0 | 2011 | 53 | 0.03 | 25.8 | 0 | 1 | 0 | 0 | 1 | 0 | 0 | 1 | 90 | 0 | 0 | 0 | 0 |
| 0 | 0 | 2011 | 53 | 0.04 | 25.8 | 0 | 0 | 1 | 0 | 1 | 0 | 1 | 0 | 87 | 0 | 0 | 0 | 1 |
| 0 | 0 | 2011 | 54 | 0.05 | 25.8 | 0 | 0 | 1 | 0 | 1 | 0 | 0 | 0 | 87 | 1 | 0 | 0 | 1 |
| 0 | 0 | 2011 | 54 | 0.05 | 25.8 | 0 | 1 | 0 | 1 | 0 | 0 | 0 | 0 | 91 | 1 | 0 | 0 | 1 |
| 0 | 0 | 2011 | 56 | 0.06 | 16.1 | 0 | 1 | 0 | 0 | 1 | 0 | 1 | 0 | 93 | 0 | 0 | 0 | 0 |
| 0 | 0 | 2011 | 54 | 0.07 | 16.1 | 0 | 1 | 0 | 0 | 1 | 0 | 1 | 0 | 96 | 0 | 0 | 0 | 0 |
| 0 | 0 | 2011 | 54 | 0.07 | 16.6 | 0 | 0 | 1 | 0 | 0 | 1 | 1 | 0 | 128 | 1 | 0 | 0 | 0 |
| 0 | 0 | 2011 | 54 | 0.07 | 16.6 | 0 | 1 | 0 | 0 | 0 | 1 | 0 | 1 | 121 | 0 | 0 | 0 | 1 |
| 0 | 0 | 2011 | 55 | 0.07 | 16.1 | 0 | 0 | 1 | 1 | 0 | 0 | 0 | 0 | 94 | 0 | 0 | 0 | 0 |
| 0 | 7 | 2011 | 37 | 0.07 | 22.1 | 1 | 0 | 0 | 1 | 0 | 0 | 0 | 0 | 109 | 0 | 1 | 0 | 1 |
| 0 | 0 | 2011 | 58 | 0.07 | 16.6 | 0 | 0 | 1 | 0 | 0 | 1 | 1 | 0 | 114 | 0 | 1 | 0 | 0 |
| 0 | 0 | 2011 | 54 | 0.07 | 16.6 | 0 | 0 | 1 | 0 | 0 | 1 | 0 | 0 | 105 | 1 | 0 | 0 | 0 |
| 0 | 0 | 2011 | 55 | 0.07 | 16.6 | 0 | 1 | 0 | 0 | 1 | 0 | 0 | 0 | 99 | 0 | 1 | 0 | 0 |
| 0 | 0 | 2011 | 52 | 0.07 | 16.1 | 0 | 1 | 0 | 0 | 0 | 1 | 1 | 0 | 107 | 1 | 0 | 0 | 0 |
| 0 | 0 | 2011 | 54 | 0.07 | 16.1 | 0 | 1 | 0 | 0 | 1 | 0 | 1 | 0 | 101 | 0 | 1 | 0 | 1 |
| 0 | 0 | 2011 | 55 | 0.08 | 16.7 | 0 | 0 | 0 | 0 | 0 | 0 | 0 | 0 | 131 | 0 | 1 | 0 | 0 |
| 0 | 3 | 2011 | 43 | 0.08 | 22.1 | 0 | 1 | 0 | 0 | 1 | 0 | 1 | 0 | 117 | 0 | 1 | 0 | 1 |
| 0 | 9 | 2011 | 54 | 0.08 | 16.6 | 0 | 1 | 0 | 0 | 1 | 0 | 0 | 0 | 88 | 0 | 1 | 0 | 0 |
| 0 | 0 | 2011 | 54 | 0.08 | 16.7 | 0 | 0 | 1 | 0 | 1 | 0 | 0 | 0 | 124 | 1 | 0 | 0 | 1 |
| 0 | 0 | 2011 | 57 | 0.08 | 16.1 | 0 | 1 | 0 | 1 | 0 | 0 | 0 | 0 | 109 | 1 | 0 | 0 | 0 |
| 0 | 0 | 2011 | 54 | 0.08 | 16.7 | 0 | 1 | 0 | 0 | 1 | 0 | 0 | 0 | 114 | 1 | 0 | 0 | 0 |
| 0 | 0 | 2011 | 58 | 0.08 | 17.6 | 1 | 0 | 0 | 0 | 1 | 0 | 0 | 0 | 105 | 1 | 0 | 0 | 1 |
| 0 | 0 | 2011 | 43 | 0.08 | 22.1 | 0 | 1 | 0 | 1 | 0 | 0 | 0 | 0 | 103 | 1 | 0 | 0 | 1 |
| 0 | 0 | 2011 | 54 | 0.08 | 16.7 | 0 | 1 | 0 | 0 | 1 | 0 | 1 | 0 | 93 | 0 | 1 | 0 | 0 |
| 0 | 0 | 2011 | 52 | 0.08 | 16.7 | 0 | 0 | 1 | 0 | 0 | 1 | 1 | 0 | 106 | 1 | 0 | 0 | 0 |
| 0 | 0 | 2011 | 55 | 0.08 | 26.0 | 0 | 1 | 0 | 0 | 1 | 0 | 0 | 1 | 89 | 1 | 0 | 0 | 0 |
| 0 | 0 | 2011 | 53 | 0.09 | 16.7 | 0 | 1 | 0 | 1 | 0 | 0 | 0 | 0 | 102 | 1 | 0 | 0 | 0 |
| 0 | 0 | 2011 | 56 | 0.09 | 26.0 | 0 | 1 | 0 | 0 | 1 | 0 | 1 | 0 | 89 | 1 | 0 | 0 | 0 |
| 0 | 0 | 2011 | 57 | 0.09 | 26.0 | 0 | 1 | 0 | 0 | 1 | 0 | 1 | 0 | 90 | 1 | 0 | 0 | 1 |
| 0 | 0 | 2011 | 57 | 0.10 | 26.0 | 0 | 1 | 0 | 1 | 0 | 0 | 0 | 0 | 104 | 1 | 0 | 0 | 1 |
| 0 | 0 | 2011 | 56 | 0.10 | 16.1 | 0 | 0 | 1 | 0 | 0 | 0 | 0 | 0 | 88 | 0 | 0 | 0 | 0 |
| 0 | 0 | 2011 | 57 | 0.11 | 16.1 | 0 | 1 | 0 | 1 | 0 | 0 | 1 | 0 | 91 | 0 | 0 | 0 | 0 |
| 0 | 0 | 2011 | 57 | 0.11 | 16.1 | 1 | 0 | 0 | 1 | 0 | 0 | 0 | 0 | 92 | 0 | 0 | 0 | 0 |
| 0 | 0 | 2011 | 57 | 0.12 | 16.1 | 0 | 1 | 0 | 0 | 1 | 0 | 1 | 0 | 95 | 0 | 0 | 0 | 0 |
| 0 | 0 | 2011 | 57 | 0.12 | 16.1 | 0 | 0 | 0 | 0 | 0 | 0 | 0 | 0 | 91 | 0 | 0 | 0 | 0 |
| 0 | 0 | 2011 | 55 | 0.12 | 16.1 | 0 | 0 | 1 | 0 | 1 | 0 | 0 | 1 | 93 | 1 | 0 | 0 | 0 |
| 0 | 0 | 2011 | 58 | 0.13 | 18.6 | 0 | 0 | 1 | 1 | 0 | 0 | 0 | 0 | 90 | 1 | 0 | 0 | 0 |
| 0 | 0 | 2011 | 54 | 0.13 | 18.6 | 0 | 0 | 1 | 0 | 1 | 0 | 1 | 0 | 92 | 0 | 0 | 0 | 0 |
| 0 | 0 | 2011 | 54 | 0.13 | 18.6 | 0 | 1 | 0 | 0 | 1 | 0 | 1 | 0 | 90 | 0 | 1 | 0 | 0 |
| 0 | 0 | 2011 | 54 | 0.14 | 18.6 | 0 | 1 | 0 | 0 | 1 | 0 | 0 | 1 | 154 | 1 | 0 | 0 | 0 |
| 0 | 0 | 2011 | 54 | 0.15 | 18.6 | 0 | 0 | 1 | 0 | 0 | 1 | 0 | 1 | 95 | 0 | 1 | 0 | 0 |
| 0 | 0 | 2011 | 53 | 0.15 | 19.4 | 0 | 0 | 1 | 0 | 0 | 1 | 0 | 1 | 100 | 1 | 0 | 0 | 0 |
| 0 | 0 | 2011 | 53 | 0.16 | 19.4 | 0 | 0 | 1 | 0 | 1 | 0 | 1 | 0 | 101 | 0 | 1 | 0 | 1 |
| 0 | 0 | 2011 | 55 | 0.17 | 19.4 | 0 | 0 | 1 | 0 | 0 | 1 | 1 | 0 | 105 | 0 | 1 | 0 | 0 |
| 0 | 0 | 2011 | 56 | 0.17 | 19.4 | 0 | 1 | 0 | 0 | 0 | 1 | 0 | 1 | 109 | 0 | 1 | 0 | 0 |
| 0 | 0 | 2011 | 54 | 0.18 | 19.4 | 0 | 1 | 0 | 0 | 0 | 1 | 1 | 0 | 116 | 0 | 1 | 0 | 1 |
| 0 | 0 | 2011 | 54 | 0.18 | 24.4 | 0 | 1 | 0 | 0 | 1 | 0 | 1 | 0 | 106 | 1 | 0 | 0 | 0 |
| 0 | 1 | 2011 | 55 | 0.19 | 24.4 | 0 | 1 | 0 | 0 | 1 | 0 | 0 | 1 | 105 | 0 | 1 | 0 | 0 |
| 0 | 0 | 2011 | 55 | 0.20 | 24.4 | 0 | 1 | 0 | 0 | 1 | 0 | 1 | 0 | 105 | 1 | 0 | 0 | 0 |
| 0 | 0 | 2011 | 57 | 0.20 | 24.4 | 1 | 0 | 0 | 0 | 0 | 0 | 0 | 0 | 99 | 0 | 1 | 0 | 0 |
| 0 | 0 | 2011 | 54 | 0.20 | 24.4 | 0 | 1 | 0 | 0 | 0 | 1 | 0 | 1 | 95 | 0 | 1 | 0 | 0 |
| 0 | 0 | 2011 | 53 | 0.21 | 24.4 | 0 | 1 | 0 | 0 | 1 | 0 | 1 | 0 | 89 | 1 | 0 | 0 | 0 |
| 0 | 0 | 2011 | 56 | 0.45 | 18.9 | 1 | 0 | 0 | 1 | 0 | 0 | 1 | 0 | 95 | 0 | 1 | 0 | 0 |
| 0 | 0 | 2011 | 56 | 0.46 | 18.9 | 0 | 1 | 0 | 0 | 1 | 0 | 0 | 1 | 99 | 0 | 1 | 0 | 0 |
| 0 | 0 | 2011 | 55 | 0.47 | 18.9 | 0 | 1 | 0 | 1 | 0 | 0 | 1 | 0 | 95 | 0 | 1 | 0 | 0 |
| 0 | 0 | 2011 | 55 | 0.47 | 18.9 | 0 | 0 | 1 | 0 | 1 | 0 | 1 | 0 | 96 | 0 | 0 | 0 | 0 |
| 0 | 0 | 2011 | 59 | 0.51 | 20.2 | 0 | 1 | 0 | 1 | 0 | 0 | 1 | 0 | 93 | 1 | 0 | 0 | 0 |
| 0 | 0 | 2011 | 52 | 0.53 | 20.2 | 0 | 1 | 0 | 0 | 1 | 0 | 1 | 0 | 91 | 0 | 0 | 0 | 0 |
| 0 | 0 | 2011 | 53 | 0.53 | 20.2 | 0 | 0 | 1 | 0 | 0 | 1 | 1 | 0 | 89 | 0 | 0 | 0 | 0 |
| 0 | 0 | 2011 | 53 | 0.53 | 20.2 | 0 | 1 | 0 | 0 | 1 | 0 | 0 | 1 | 89 | 1 | 0 | 0 | 0 |
| 0 | 0 | 2011 | 20 | 0.54 | 28.0 | 0 | 0 | 1 | 1 | 0 | 0 | 0 | 0 | 95 | 1 | 0 | 0 | 1 |
| 0 | 0 | 2011 | 20 | 0.54 | 28.0 | 0 | 1 | 0 | 1 | 0 | 0 | 0 | 0 | 92 | 0 | 1 | 0 | 1 |
| 0 | 0 | 2011 | 21 | 0.54 | 28.0 | 0 | 0 | 1 | 0 | 0 | 1 | 1 | 0 | 96 | 0 | 1 | 0 | 1 |
| 0 | 0 | 2011 | 20 | 0.55 | 28.0 | 0 | 1 | 0 | 1 | 0 | 0 | 1 | 0 | 92 | 1 | 0 | 0 | 1 |
| 0 | 0 | 2011 | 20 | 0.55 | 28.0 | 0 | 0 | 1 | 0 | 0 | 1 | 0 | 1 | 87 | 1 | 0 | 0 | 1 |
| 0 | 1 | 2011 | 21 | 0.55 | 28.0 | 0 | 1 | 0 | 1 | 0 | 0 | 0 | 0 | 96 | 1 | 0 | 0 | 1 |
| 0 | 0 | 2011 | 38 | 0.57 | 22.3 | 1 | 0 | 0 | 1 | 0 | 0 | 0 | 0 | 98 | 1 | 0 | 0 | 0 |
| 0 | 3 | 2011 | 38 | 0.57 | 22.3 | 0 | 1 | 0 | 0 | 1 | 0 | 0 | 1 | 100 | 0 | 1 | 0 | 0 |
| 0 | 0 | 2011 | 38 | 0.57 | 22.3 | 1 | 0 | 0 | 0 | 1 | 0 | 1 | 0 | 95 | 1 | 0 | 0 | 0 |
| 0 | 1 | 2011 | 36 | 0.57 | 22.3 | 0 | 1 | 0 | 0 | 1 | 0 | 1 | 0 | 90 | 1 | 0 | 0 | 0 |
| 0 | 0 | 2011 | 37 | 0.58 | 22.3 | 0 | 1 | 0 | 0 | 1 | 0 | 1 | 0 | 84 | 0 | 1 | 0 | 0 |
| 0 | 0 | 2011 | 38 | 0.58 | 22.3 | 1 | 0 | 0 | 1 | 0 | 0 | 0 | 0 | 87 | 1 | 0 | 0 | 0 |
| 0 | 0 | 2011 | 54 | 0.61 | 19.4 | 0 | 0 | 1 | 0 | 0 | 1 | 0 | 0 | 91 | 1 | 0 | 0 | 0 |
| 0 | 0 | 2011 | 51 | 0.62 | 19.4 | 0 | 1 | 0 | 0 | 0 | 1 | 1 | 0 | 94 | 1 | 0 | 0 | 0 |
| 0 | 0 | 2011 | 51 | 0.62 | 19.4 | 0 | 1 | 0 | 0 | 1 | 0 | 0 | 0 | 94 | 1 | 0 | 0 | 0 |
| 0 | 0 | 2011 | 51 | 0.63 | 19.4 | 1 | 0 | 0 | 0 | 1 | 0 | 0 | 1 | 97 | 1 | 0 | 0 | 0 |
| 0 | 0 | 2011 | 54 | 0.63 | 19.4 | 0 | 0 | 1 | 0 | 1 | 0 | 1 | 0 | 98 | 0 | 0 | 0 | 0 |
| 0 | 0 | 2011 | 58 | 0.63 | 19.4 | 0 | 1 | 0 | 0 | 1 | 0 | 1 | 0 | 100 | 0 | 1 | 0 | 0 |
| 0 | 1 | 2011 | 21 | 0.65 | 28.6 | 0 | 1 | 0 | 0 | 1 | 0 | 1 | 0 | 93 | 1 | 0 | 0 | 1 |
| 0 | 0 | 2011 | 21 | 0.65 | 28.6 | 0 | 1 | 0 | 1 | 0 | 0 | 0 | 0 | 99 | 1 | 0 | 0 | 1 |
| 0 | 0 | 2011 | 23 | 0.66 | 28.6 | 0 | 0 | 1 | 0 | 0 | 1 | 0 | 1 | 109 | 0 | 1 | 0 | 1 |
| 0 | 0 | 2011 | 21 | 0.66 | 28.6 | 0 | 1 | 0 | 1 | 0 | 0 | 0 | 0 | 104 | 0 | 1 | 0 | 1 |
| 0 | 0 | 2011 | 22 | 0.66 | 28.6 | 0 | 1 | 0 | 0 | 1 | 0 | 0 | 1 | 104 | 1 | 0 | 0 | 1 |
| 0 | 1 | 2011 | 21 | 0.66 | 28.6 | 1 | 0 | 0 | 0 | 1 | 0 | 1 | 0 | 99 | 1 | 0 | 0 | 1 |
| 0 | 0 | 2011 | 45 | 0.67 | 21.8 | 0 | 0 | 1 | 0 | 1 | 0 | 0 | 0 | 85 | 0 | 1 | 0 | 0 |
| 0 | 0 | 2011 | 43 | 0.67 | 21.8 | 0 | 1 | 0 | 0 | 1 | 0 | 0 | 0 | 87 | 1 | 0 | 0 | 0 |
| 0 | 0 | 2011 | 44 | 0.67 | 21.8 | 0 | 0 | 1 | 0 | 0 | 1 | 0 | 0 | 91 | 0 | 0 | 0 | 0 |
| 0 | 0 | 2011 | 42 | 0.68 | 21.8 | 0 | 0 | 1 | 0 | 1 | 0 | 1 | 0 | 93 | 0 | 0 | 0 | 0 |
| 0 | 0 | 2011 | 43 | 0.68 | 21.8 | 0 | 0 | 0 | 1 | 0 | 0 | 1 | 0 | 95 | 0 | 1 | 0 | 0 |
| 0 | 0 | 2011 | 43 | 0.68 | 21.8 | 0 | 1 | 0 | 1 | 0 | 0 | 1 | 0 | 98 | 1 | 0 | 0 | 0 |
| 0 | 0 | 2011 | 44 | 0.76 | 22.7 | 0 | 1 | 0 | 1 | 0 | 0 | 0 | 0 | 85 | 1 | 0 | 0 | 0 |
| 0 | 0 | 2011 | 45 | 0.77 | 22.7 | 0 | 1 | 0 | 1 | 0 | 0 | 0 | 0 | 88 | 0 | 0 | 0 | 0 |
| 0 | 0 | 2011 | 43 | 0.77 | 22.7 | 1 | 0 | 0 | 0 | 1 | 0 | 0 | 0 | 94 | 0 | 1 | 0 | 0 |
| 0 | 0 | 2011 | 43 | 0.78 | 22.7 | 0 | 1 | 0 | 1 | 0 | 0 | 0 | 0 | 96 | 0 | 0 | 0 | 0 |
| 0 | 0 | 2011 | 35 | 0.90 | 24.0 | 1 | 0 | 0 | 0 | 0 | 0 | 0 | 0 | 92 | 1 | 0 | 0 | 0 |
| 0 | 0 | 2011 | 36 | 0.90 | 24.0 | 1 | 0 | 0 | 1 | 0 | 0 | 0 | 0 | 95 | 1 | 0 | 1 | 0 |
| 0 | 0 | 2011 | 35 | 0.91 | 24.0 | 1 | 0 | 0 | 1 | 0 | 0 | 0 | 0 | 92 | 0 | 1 | 0 | 0 |
| 0 | 0 | 2011 | 35 | 0.91 | 24.0 | 1 | 0 | 0 | 1 | 0 | 0 | 0 | 0 | 94 | 0 | 0 | 0 | 1 |
| 0 | 0 | 2011 | 34 | 0.91 | 24.0 | 1 | 0 | 0 | 1 | 0 | 0 | 0 | 0 | 91 | 1 | 0 | 1 | 1 |
| 0 | 0 | 2011 | 35 | 0.91 | 24.0 | 0 | 0 | 0 | 1 | 0 | 0 | 0 | 0 | 94 | 1 | 0 | 0 | 1 |
| 0 | 0 | 2011 | 49 | 1.02 | 21.8 | 1 | 0 | 0 | 1 | 0 | 0 | 0 | 0 | 88 | 0 | 0 | 0 | 1 |
| 0 | 0 | 2011 | 49 | 1.03 | 21.8 | 0 | 1 | 0 | 1 | 0 | 0 | 0 | 0 | 84 | 1 | 0 | 0 | 1 |
| 0 | 0 | 2011 | 50 | 1.03 | 21.8 | 0 | 1 | 0 | 1 | 0 | 0 | 0 | 0 | 84 | 0 | 1 | 0 | 1 |
| 0 | 0 | 2011 | 50 | 1.04 | 21.8 | 1 | 0 | 0 | 1 | 0 | 0 | 0 | 0 | 89 | 0 | 0 | 0 | 1 |
| 0 | 0 | 2011 | 50 | 1.04 | 21.8 | 1 | 0 | 0 | 0 | 1 | 0 | 1 | 0 | 92 | 0 | 1 | 0 | 1 |
| 0 | 0 | 2011 | 50 | 1.04 | 21.8 | 1 | 0 | 0 | 1 | 0 | 0 | 0 | 0 | 94 | 0 | 1 | 0 | 1 |
| 0 | 0 | 2011 | 53 | 1.07 | 20.3 | 0 | 0 | 1 | 0 | 0 | 0 | 0 | 0 | 82 | 1 | 0 | 1 | 0 |
| 0 | 0 | 2011 | 55 | 1.07 | 20.3 | 0 | 1 | 0 | 0 | 0 | 1 | 0 | 1 | 78 | 1 | 0 | 1 | 0 |
| 0 | 0 | 2011 | 51 | 1.08 | 20.3 | 1 | 0 | 0 | 0 | 0 | 0 | 0 | 0 | 85 | 1 | 0 | 1 | 0 |
| 0 | 0 | 2011 | 53 | 1.08 | 20.3 | 0 | 1 | 0 | 0 | 1 | 0 | 1 | 0 | 92 | 1 | 0 | 1 | 0 |
| 0 | 0 | 2011 | 53 | 1.09 | 20.3 | 1 | 0 | 0 | 1 | 0 | 0 | 1 | 0 | 90 | 1 | 0 | 1 | 0 |
| 0 | 0 | 2011 | 49 | 1.09 | 20.3 | 0 | 0 | 0 | 0 | 0 | 0 | 0 | 0 | 91 | 1 | 0 | 1 | 0 |
| 0 | 0 | 2011 | 52 | 1.09 | 20.6 | 0 | 0 | 1 | 0 | 1 | 0 | 1 | 0 | 91 | 0 | 0 | 0 | 1 |
| 0 | 0 | 2011 | 53 | 1.10 | 20.6 | 0 | 0 | 1 | 0 | 1 | 0 | 0 | 1 | 91 | 0 | 1 | 0 | 1 |
| 0 | 0 | 2011 | 53 | 1.10 | 20.6 | 0 | 1 | 0 | 0 | 1 | 0 | 1 | 0 | 93 | 0 | 0 | 1 | 1 |
| 0 | 0 | 2011 | 53 | 1.11 | 20.6 | 0 | 0 | 1 | 1 | 0 | 0 | 0 | 0 | 95 | 1 | 0 | 1 | 1 |
| 0 | 0 | 2011 | 49 | 1.12 | 20.6 | 0 | 1 | 0 | 0 | 1 | 0 | 1 | 0 | 96 | 0 | 0 | 1 | 1 |
| 1 | 0 | 2011 | 50 | 1.17 | 27.4 | 1 | 0 | 0 | 0 | 1 | 0 | 0 | 0 | 105 | 0 | 1 | 0 | 0 |
| 0 | 0 | 2011 | 49 | 1.17 | 22.2 | 0 | 1 | 0 | 0 | 1 | 0 | 1 | 0 | 87 | 1 | 0 | 1 | 0 |
| 0 | 0 | 2011 | 50 | 1.17 | 27.4 | 1 | 0 | 0 | 1 | 0 | 0 | 0 | 0 | 99 | 1 | 0 | 0 | 0 |
| 0 | 1 | 2011 | 50 | 1.17 | 22.2 | 1 | 0 | 0 | 0 | 1 | 0 | 1 | 0 | 87 | 1 | 0 | 1 | 0 |
| 0 | 5 | 2011 | 50 | 1.18 | 27.4 | 0 | 1 | 0 | 0 | 1 | 0 | 0 | 0 | 94 | 0 | 1 | 0 | 0 |
| 0 | 0 | 2011 | 48 | 1.18 | 22.2 | 0 | 1 | 0 | 0 | 1 | 0 | 1 | 0 | 88 | 0 | 0 | 0 | 0 |
| 0 | 0 | 2011 | 51 | 1.18 | 27.4 | 1 | 0 | 0 | 1 | 0 | 0 | 0 | 0 | 91 | 0 | 1 | 0 | 0 |
| 0 | 0 | 2011 | 50 | 1.19 | 22.1 | 0 | 0 | 1 | 0 | 0 | 0 | 0 | 0 | 93 | 1 | 0 | 0 | 0 |
| 0 | 0 | 2011 | 50 | 1.19 | 22.1 | 0 | 0 | 1 | 1 | 0 | 0 | 0 | 0 | 95 | 0 | 0 | 0 | 0 |
| 0 | 0 | 2011 | 48 | 1.20 | 22.1 | 0 | 0 | 1 | 0 | 1 | 0 | 0 | 1 | 98 | 0 | 1 | 0 | 0 |
| 0 | 0 | 2011 | 51 | 1.20 | 22.1 | 0 | 0 | 1 | 1 | 0 | 0 | 0 | 0 | 98 | 0 | 1 | 0 | 0 |
| 0 | 1 | 2011 | 38 | 1.21 | 24.5 | 1 | 0 | 0 | 0 | 1 | 0 | 0 | 0 | 80 | 1 | 0 | 0 | 0 |
| 1 | 1 | 2011 | 38 | 1.21 | 24.5 | 0 | 1 | 0 | 0 | 1 | 0 | 1 | 0 | 87 | 1 | 0 | 0 | 0 |
| 0 | 0 | 2011 | 54 | 1.21 | 22.1 | 0 | 1 | 0 | 0 | 1 | 0 | 1 | 0 | 98 | 1 | 0 | 0 | 0 |
| 1 | 10 | 2011 | 38 | 1.21 | 24.5 | 0 | 1 | 0 | 0 | 0 | 1 | 1 | 0 | 77 | 1 | 0 | 0 | 1 |
| 0 | 3 | 2011 | 33 | 1.21 | 28.0 | 0 | 0 | 0 | 0 | 0 | 0 | 0 | 0 | 90 | 1 | 0 | 0 | 1 |
| 0 | 0 | 2011 | 53 | 1.21 | 22.1 | 0 | 1 | 0 | 1 | 0 | 0 | 0 | 0 | 97 | 0 | 0 | 0 | 0 |
| 0 | 0 | 2011 | 36 | 1.21 | 28.0 | 0 | 0 | 0 | 1 | 0 | 0 | 0 | 0 | 104 | 0 | 1 | 0 | 1 |
| 0 | 0 | 2011 | 38 | 1.21 | 24.5 | 0 | 1 | 0 | 0 | 1 | 0 | 1 | 0 | 85 | 1 | 0 | 0 | 1 |
| 0 | 4 | 2011 | 33 | 1.22 | 28.0 | 1 | 0 | 0 | 1 | 0 | 0 | 0 | 0 | 97 | 0 | 1 | 0 | 1 |
| 0 | 0 | 2011 | 38 | 1.35 | 21.3 | 1 | 0 | 0 | 1 | 0 | 0 | 0 | 0 | 88 | 1 | 0 | 0 | 0 |
| 0 | 2 | 2011 | 36 | 1.36 | 21.3 | 0 | 1 | 0 | 0 | 1 | 0 | 0 | 0 | 83 | 1 | 0 | 0 | 0 |
| 0 | 0 | 2011 | 36 | 1.36 | 21.3 | 1 | 0 | 0 | 1 | 0 | 0 | 0 | 0 | 80 | 1 | 0 | 0 | 0 |
| 0 | 0 | 2011 | 36 | 1.36 | 21.3 | 0 | 1 | 0 | 0 | 1 | 0 | 1 | 0 | 88 | 1 | 0 | 0 | 0 |
| 0 | 0 | 2011 | 36 | 1.36 | 21.3 | 1 | 0 | 0 | 1 | 0 | 0 | 0 | 0 | 92 | 1 | 0 | 0 | 0 |
| 0 | 0 | 2011 | 37 | 1.36 | 21.3 | 1 | 0 | 0 | 1 | 0 | 0 | 0 | 0 | 87 | 1 | 0 | 0 | 0 |
| 0 | 0 | 2011 | 66 | 1.39 | 20.8 | 1 | 0 | 0 | 0 | 1 | 0 | 0 | 0 | 86 | 1 | 0 | 0 | 1 |
| 0 | 0 | 2011 | 62 | 1.39 | 20.8 | 0 | 1 | 0 | 0 | 1 | 0 | 0 | 0 | 93 | 0 | 1 | 0 | 1 |
| 0 | 0 | 2011 | 71 | 1.40 | 20.8 | 0 | 0 | 0 | 1 | 0 | 0 | 0 | 0 | 103 | 0 | 0 | 1 | 1 |
| 0 | 0 | 2011 | 69 | 1.40 | 20.8 | 0 | 1 | 0 | 1 | 0 | 0 | 0 | 0 | 97 | 0 | 0 | 0 | 1 |
| 0 | 0 | 2011 | 72 | 1.40 | 20.4 | 0 | 0 | 0 | 0 | 0 | 0 | 0 | 0 | 110 | 1 | 0 | 0 | 1 |
| 0 | 0 | 2011 | 68 | 1.40 | 20.8 | 0 | 0 | 0 | 1 | 0 | 0 | 0 | 0 | 105 | 0 | 0 | 1 | 1 |
| 0 | 0 | 2011 | 71 | 1.41 | 20.4 | 1 | 0 | 0 | 1 | 0 | 0 | 0 | 0 | 100 | 0 | 0 | 0 | 1 |
| 0 | 1 | 2011 | 69 | 1.41 | 20.4 | 1 | 0 | 0 | 1 | 0 | 0 | 0 | 0 | 100 | 0 | 1 | 0 | 1 |
| 0 | 0 | 2011 | 70 | 1.41 | 20.4 | 0 | 1 | 0 | 1 | 0 | 0 | 0 | 0 | 94 | 0 | 0 | 0 | 1 |
| 0 | 0 | 2011 | 70 | 1.42 | 20.4 | 0 | 1 | 0 | 0 | 1 | 0 | 1 | 0 | 88 | 1 | 0 | 0 | 1 |
| 0 | 0 | 2011 | 72 | 1.42 | 20.4 | 1 | 0 | 0 | 1 | 0 | 0 | 0 | 0 | 85 | 0 | 1 | 0 | 0 |
| 0 | 1 | 2011 | 57 | 1.43 | 23.1 | 1 | 0 | 0 | 1 | 0 | 0 | 0 | 0 | 91 | 0 | 0 | 0 | 0 |
| 1 | 0 | 2011 | 59 | 1.44 | 23.1 | 1 | 0 | 0 | 0 | 1 | 0 | 0 | 0 | 94 | 0 | 0 | 0 | 0 |
| 0 | 0 | 2011 | 58 | 1.44 | 23.1 | 1 | 0 | 0 | 1 | 0 | 0 | 0 | 0 | 97 | 0 | 0 | 0 | 0 |
| 0 | 1 | 2011 | 60 | 1.44 | 23.1 | 0 | 1 | 0 | 0 | 1 | 0 | 0 | 0 | 97 | 0 | 1 | 0 | 1 |
| 0 | 0 | 2011 | 60 | 1.45 | 23.1 | 1 | 0 | 0 | 1 | 0 | 0 | 0 | 0 | 99 | 1 | 0 | 0 | 0 |
| 0 | 0 | 2011 | 32 | 1.47 | 28.8 | 1 | 0 | 0 | 1 | 0 | 0 | 0 | 0 | 115 | 1 | 0 | 0 | 1 |
| 0 | 0 | 2011 | 32 | 1.47 | 28.8 | 1 | 0 | 0 | 1 | 0 | 0 | 0 | 0 | 114 | 1 | 0 | 0 | 1 |
| 0 | 0 | 2011 | 31 | 1.48 | 28.8 | 0 | 1 | 0 | 0 | 1 | 0 | 1 | 0 | 114 | 0 | 0 | 0 | 1 |
| 0 | 0 | 2011 | 32 | 1.48 | 28.8 | 1 | 0 | 0 | 1 | 0 | 0 | 0 | 0 | 97 | 1 | 0 | 0 | 1 |
| 0 | 0 | 2011 | 30 | 1.48 | 28.8 | 0 | 1 | 0 | 0 | 1 | 0 | 1 | 0 | 102 | 1 | 0 | 0 | 1 |
| 0 | 0 | 2011 | 31 | 1.48 | 28.8 | 0 | 1 | 0 | 0 | 1 | 0 | 0 | 0 | 99 | 0 | 1 | 0 | 1 |
| 0 | 0 | 2011 | 30 | 1.48 | 28.8 | 1 | 0 | 0 | 0 | 0 | 0 | 0 | 0 | 110 | 0 | 0 | 0 | 1 |
| 0 | 3 | 2011 | 31 | 1.48 | 28.8 | 0 | 1 | 0 | 0 | 1 | 0 | 1 | 0 | 106 | 1 | 0 | 0 | 1 |
| 0 | 0 | 2011 | 31 | 1.49 | 28.8 | 0 | 0 | 0 | 0 | 0 | 0 | 0 | 0 | 112 | 1 | 0 | 0 | 1 |
| 0 | 1 | 2011 | 68 | 1.50 | 23.3 | 1 | 0 | 0 | 1 | 0 | 0 | 0 | 0 | 98 | 0 | 1 | 0 | 1 |
| 0 | 0 | 2011 | 73 | 1.50 | 23.3 | 1 | 0 | 0 | 1 | 0 | 0 | 0 | 0 | 96 | 0 | 1 | 0 | 1 |
| 0 | 0 | 2011 | 71 | 1.51 | 23.3 | 1 | 0 | 0 | 0 | 0 | 0 | 0 | 0 | 105 | 1 | 0 | 0 | 1 |
| 0 | 1 | 2011 | 70 | 1.51 | 23.3 | 1 | 0 | 0 | 1 | 0 | 0 | 0 | 0 | 93 | 1 | 0 | 0 | 1 |
| 1 | 0 | 2011 | 70 | 1.51 | 23.3 | 1 | 0 | 0 | 1 | 0 | 0 | 0 | 0 | 90 | 0 | 1 | 0 | 0 |
| 1 | 5 | 2011 | 71 | 1.52 | 23.6 | 1 | 0 | 0 | 1 | 0 | 0 | 0 | 0 | 108 | 1 | 0 | 0 | 1 |
| 0 | 0 | 2011 | 73 | 1.52 | 23.6 | 1 | 0 | 0 | 1 | 0 | 0 | 0 | 0 | 100 | 1 | 0 | 0 | 1 |
| 0 | 0 | 2011 | 75 | 1.52 | 23.6 | 0 | 0 | 0 | 1 | 0 | 0 | 0 | 0 | 91 | 1 | 0 | 0 | 1 |
| 0 | 1 | 2011 | 68 | 1.53 | 23.6 | 0 | 1 | 0 | 1 | 0 | 0 | 0 | 0 | 111 | 1 | 0 | 0 | 1 |
| 0 | 6 | 2011 | 66 | 1.53 | 23.6 | 1 | 0 | 0 | 1 | 0 | 0 | 0 | 0 | 111 | 1 | 0 | 0 | 0 |
| 1 | 2 | 2011 | 67 | 1.53 | 23.6 | 0 | 1 | 0 | 1 | 0 | 0 | 0 | 0 | 115 | 0 | 1 | 0 | 1 |
| 0 | 0 | 2011 | 30 | 1.57 | 27.4 | 0 | 1 | 0 | 0 | 0 | 0 | 0 | 0 | 103 | 0 | 0 | 0 | 1 |
| 0 | 0 | 2011 | 30 | 1.57 | 27.4 | 0 | 1 | 0 | 0 | 0 | 0 | 0 | 0 | 107 | 0 | 0 | 0 | 1 |
| 0 | 0 | 2011 | 30 | 1.57 | 27.4 | 1 | 0 | 0 | 0 | 0 | 0 | 0 | 0 | 101 | 0 | 0 | 0 | 1 |
| 0 | 0 | 2011 | 31 | 1.57 | 27.4 | 0 | 1 | 0 | 0 | 0 | 0 | 0 | 0 | 98 | 0 | 1 | 0 | 1 |
| 1 | 0 | 2011 | 30 | 1.57 | 27.4 | 1 | 0 | 0 | 1 | 0 | 0 | 0 | 0 | 91 | 1 | 0 | 0 | 1 |
| 0 | 0 | 2011 | 30 | 1.57 | 27.4 | 1 | 0 | 0 | 0 | 0 | 0 | 0 | 0 | 94 | 0 | 1 | 0 | 1 |
| 0 | 0 | 2011 | 34 | 1.61 | 27.6 | 1 | 0 | 0 | 0 | 1 | 0 | 0 | 1 | 92 | 0 | 1 | 0 | 0 |
| 0 | 0 | 2011 | 32 | 1.61 | 27.6 | 1 | 0 | 0 | 1 | 0 | 0 | 0 | 0 | 91 | 1 | 0 | 0 | 0 |
| 0 | 0 | 2011 | 34 | 1.61 | 27.6 | 1 | 0 | 0 | 1 | 0 | 0 | 0 | 0 | 93 | 1 | 0 | 0 | 0 |
| 0 | 6 | 2011 | 34 | 1.61 | 27.6 | 1 | 0 | 0 | 0 | 1 | 0 | 1 | 0 | 91 | 1 | 0 | 0 | 0 |
