# Supplementary material for: Occupancy Models for Monitoring Marine Fish: A Bayesian Hierarchical Approach to Model Imperfect Detection with a Novel Gear Combination
Source: PLoS One. 2014 Sep 25;9(9):e108302. doi: 10.1371/journal.pone.0108302 (PMC4178000; doi:10.1371/journal.pone.0108302)
Supplement: Appendix S2 — JAGS code for Royle and Nichols (2003) occupancy model of red snapper abundance and detection with pooled camera trap detections. The “#” symbol precedes annotation remarks. Refer to the Methods section of the main text for symbol equations and parameter definitions. (DOCX) [file pone.0108302.s003.docx]

model {

#Priors

for(i in 1:16){

bet[i] ~ dnorm(0,0.01)

}

for(i in 1:7){

alpha[i] ~ dt(0,pow(1.566267,-2),7.63179)

}

for(i in 1:5){

phi[i] ~ dt(0,pow(1.566267,-2),7.63179)

}

#Abundance model

for(i in 1:n){

N[i] ~ dpois(lambda[i])

lambda [i] <- exp(log.lambda[i])

log.lambda[i] <- bet[1] + bet[2]*year[i] + bet[3]*depth[i,1] + bet[4]*depth2[i,1] +

bet[5]*lat[i,1] + bet[6]*lat2[i,1] + bet[7]*temp[i,1] + bet[8]*temp2[i,1] +

bet[9]*livebot.l[i] + bet[10]*livebot.m[i] + w[10]*bet[11]*livebot.h[i] +

bet[12]*hardsub.l[i] + bet[13]*hardsub.m[i] + bet[14]*hardsub.h[i] +

bet[15]*relief.m[i] + bet[16]*relief.h[i]

#Detection model (Chevron trap)

y[i,1] ~ dbern(p[i,1])

p[i,1] <- 1-(1-r[i,1])^N[i]

r[i,1] <- exp(logit.r[i,1])/(1+exp(logit.r[i,1]))

logit.r[i,1] <- alpha[1] + alpha[2]*temp[i,1] + alpha[3]*temp2[i,1] + alpha[4]*soak[i,1] +

alpha[5]*cdir.p[i,2] + alpha[6]*cdir.a[i,1] + alpha[7]*cspeed[i,1]

#Detection model (Camera trap)

y[i,2] ~ dbern(p[i,2])

p[i,2] <- 1-(1-r[i,2])^N[i]

r[i,2] <- exp(logit.r[i,2])/(1+exp(logit.r[i,2]))

logit.r[i,2] <- phi[1] + phi[2]*turb[i,2] + phi[3]*cdir.p[i,2] + phi[4]*cdir.a[i,2] +

phi[5]*cspeed[i,2]

} #end loop i

}
